# OTUD6A‐Mediated Deubiquitination of PRDX1 Protects Against Oral Ulcer by Restoring Mitochondrial Function

**DOI:** 10.1002/advs.77077

**Published:** 2026-08-12

**Authors:** Xiaoyu Sun, Ruiwei Jia, Xingbei Pan, Rui Huang, Jiahong Chen, Ziyi Yao, Lei Yin, Jun Ma, Hongjieliang Wang, Xinyu Huang, Yilin Ma, Yifan Ping, Yuanyuan Chen, Huining Wang, Shengbin Huang, Guang Liang

**Affiliations:** ^1^ Institute of Stomatology School and Hospital of Stomatology Wenzhou Medical University Wenzhou Zhejiang China; ^2^ Department of Periodontics School and Hospital of Stomatology Wenzhou Medical University Wenzhou Zhejiang China; ^3^ Department of Prosthodontics School and Hospital of Stomatology Wenzhou Medical University Wenzhou Zhejiang China; ^4^ Chemical Biology Research Center School of Pharmaceutical Sciences Wenzhou Medical University Wenzhou Zhejiang China; ^5^ School of Pharmaceutical Sciences Hangzhou Medical College Hangzhou Zhejiang China

**Keywords:** deubiquitinating enzyme, mitochondrial dysfunction, oral ulcer (OU), OTU domain‐containing protein 6A (OTUD6A), peroxiredoxin‐1 (PRDX1)

## Abstract

Oral ulcers (OU), as the most highly prevalent and recurrent oral mucosal lesion, have an unclear pathogenesis that hampers the development of effective treatments. While emerging evidence suggests that deubiquitinating enzymes (DUBs) may play a role in oral diseases, their functions in OU remain unclear. In this study, we screened the expression of DUBs in tongue tissues from mice with OU and identified OTU domain‐containing protein 6A (OTUD6A) as the most substantially downregulated DUB during OU progression. The functional studies demonstrated that *OTUD6A* deficiency exacerbated OU pathology in mice and, correspondingly, suppressed proliferation and migration while promoting apoptosis of human oral keratinocytes (HOKs). Mechanistic investigations revealed that OTUD6A directly bound to peroxiredoxin‐1 (PRDX1) and cleaved its K48‐linked polyubiquitin chains at lysine 136 site (K136) via the catalytic residue C152, thereby stabilizing PRDX1 in HOKs. Loss of OTUD6A led to PRDX1 degradation, which enhanced mitochondrial oxidative stress and dysfunction, ultimately impairing HOKs function, while *OTUD6A* overexpression rescued HOKs malfunction. Importantly, local administration of recombinant OTUD6A protein significantly accelerated OU healing in mice. Collectively, these results identify a new OTUD6A‐PRDX1 axis in OU pathology and present OTUD6A as a promising therapeutic target for OU.

## Graphical abstract

In human oral keratinocytes, OTUD6A promoted cell migration by deubiquitinating and stabilizing PRDX1. Conversely, OTUD6A deficiency reduced PRDX1 stability, triggering mitochondrial dysfunction and aggravating OU progression.

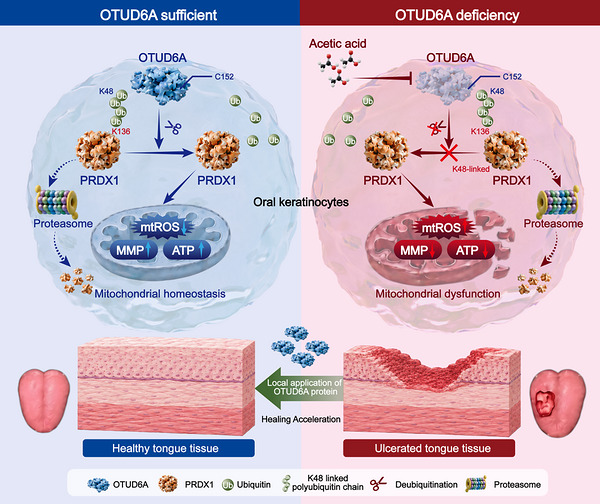

## 1 Introduction

Affecting over 25% of the population, oral ulcers (OU) are the most common and painful oral mucosal conditions, and also a common complication of radiotherapy and/or chemotherapy for patients with cancer [1, 2]. Pathologically, OU present as round or oval defects with epithelial discontinuity, inflammatory infiltration, and a gray‐white pseudomembrane. The highly innervated nature of the oral mucosa results in significant pain, which impairs chewing, swallowing, and overall quality of life [3, 4]. The etiology of OU is multifactorial and remains incompletely understood, involving traumatic, infectious, and allergic factors, and may be associated with skin diseases, autoimmune diseases, tumors, inflammatory bowel diseases, and so on. However, in many cases, including recurrent aphthous ulcers, the exact cause is unconfirmed [5, 6]. Current OU management relies on topical corticosteroids, systemic immunomodulators, and analgesics, which primarily provide symptomatic relief. There has been no established effective therapy [6, 7, 8]. The lack of definitive diagnostic biomarkers and targeted therapies highlights an urgent need to unravel the core pathological mechanisms driving OU to identify more effective treatment.

Ubiquitination is a key post‐translational modification that regulates protein stability and function through the covalent attachment of ubiquitin [9]. This reversible process is precisely counterbalanced by deubiquitinating enzymes (DUBs), which cleave ubiquitin conjugates. By modulating protein stability and function, DUBs shape cellular phenotypes and influence pathogenesis of various diseases [10], making them promising therapeutic targets for inflammatory, metabolic, and immune disorders, including diabetes and sepsis [11]. A growing body of evidence has linked specific deubiquitinating enzymes, such as OTUD1, A20, CYLD, and UCH‐L1, to the pathogenesis of periodontitis [12]. Notable studies further indicated that targeted modulation of DUB activity attenuated periodontitis progression [8, 13]. Separately, USP9X has been implicated in head and neck cancer [14]. Nonetheless, research on oral diseases has predominantly focused on periodontitis and head and neck cancer, leaving other oral conditions significantly underexplored. In particular, the specific DUB governing OU pathogenesis remains unidentified.

The DUB superfamily comprises six major families, with the ovarian tumor domain‐containing (OTU) protease family being the largest and most critical class [15]. To identify the potential involvement of DUBs in OU pathology, we focused initially on the OTU family. Quantitative real‐time polymerase chain reaction (qRT‐PCR) analysis of OTU family genes revealed that OTU domain‐containing protein 6A (OTUD6A) was the most significantly downregulated DUB during OU progression, implicating it as a potential contributor to OU pathogenesis. OTUD6A is a recently characterized deubiquitinase that modulates innate immune responses and inflammatory signaling pathways, with well‐documented roles in cancer, immune, and other inflammatory disorders [16, 17, 18, 19]. Nevertheless, how OTUD6A specifically regulates OU pathology and its underlying mechanisms remains unclear.

In this study, we demonstrated that OTUD6A deficiency exacerbated OU progression, whereas supplementation with OTUD6A protein accelerated OU healing in mice. In vitro, OTUD6A protected human oral keratinocytes (HOKs) by simultaneously suppressing apoptosis and promoting proliferation. Mechanistically, OTUD6A directly bound to peroxiredoxin‐1 (PRDX1), a key antioxidative enzyme, and catalyzed its deubiquitination, thereby stabilizing PRDX1 and protecting HOKs from oxidative stress and mitochondrial damage during OU. These findings establish the OTUD6A–PRDX1–mitochondrial axis as a critical regulatory pathway in OU pathogenesis and highlight OTUD6A as a promising therapeutic target.

## 2 Results

### 2.1 OTUD6A Deficiency Aggravated the Tissue Destruction of OU

**FIGURE 1:**
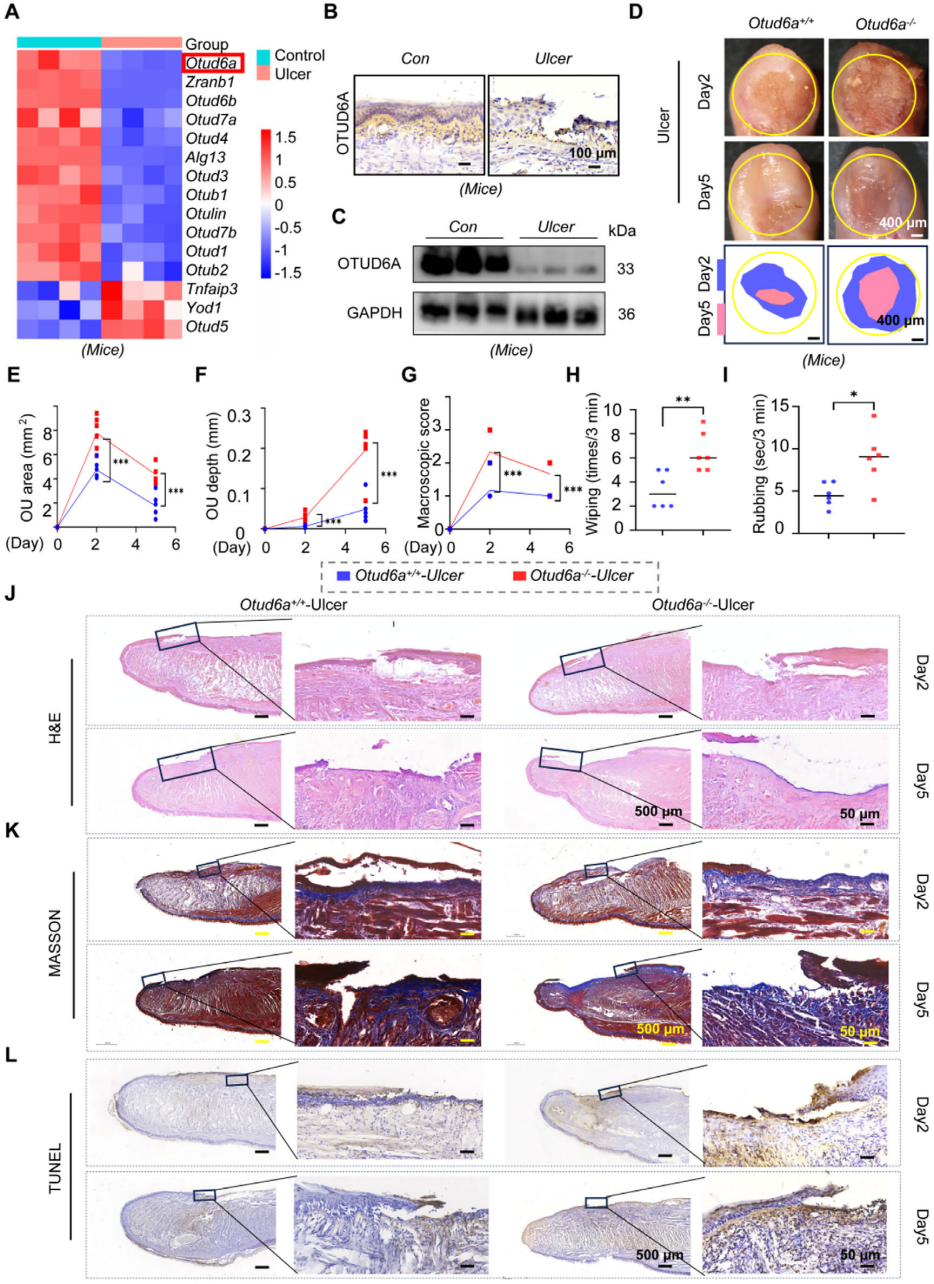
OTUD6A deficiency aggravated the symptoms of OUs. (A) qRT‐PCR analysis on both ulcerated and normal tongue tissues from mice. (B, C) Representative immunohistochemistry image and immunoblot of OTUD6A protein from mice with or without OU, n = 6 per group. Scale bar = 100 µm. (D) Typical image and wound overlay image of tongues of *Otud6a^+/+^* mice and *Otud6a^−/−^* mice on day 2 and day 5, n = 6 per group. Scale bar = 400 µm. (E) Quantitative assessment of the area of OU. Data are presented as mean ± SEM, n = 6 per group. (F) Quantitative assessment of the depth of OU. Data are presented as mean ± SEM, n = 6 per group. (G) Quantitative assessment of macroscopic scores evaluating the clinical severity of OU. Data are presented as mean ± SEM, n = 6 per group. (H) Quantitative assessment of the number of facial wiping and (I) Quantitative assessment of rubbing time during a 3‐min block of OU mice. Data are presented as mean ± SEM, n = 6 per group. (J) Hematoxylin and eosin (H&E) staining of *Otud6a^+/+^* mice and *Otud6a^−/−^* mice. n = 6 per group. Scale bar = 500 µm (Enlarged image scale bar = 50 µm). (K) Masson trichrome stain of *Otud6a* ^+/+^ mice and *Otud6a^−/−^* mice. n = 6 per group. Scale bar = 500 µm (Enlarged image scale bar = 50 µm). (L) TUNEL staining of *Otud6a^+/+^* and *Otud6a^−/−^* mice. Data are presented as mean ± SEM, n = 6 per group. Scale bar = 500 µm (Enlarged image scale bar = 50 µm). ^*^p < 0.05, ^**^p < 0.01, ^***^p < 0.001.

To investigate the involvement of OTU family members in OU, we performed qRT‐PCR on both ulcerated and normal tongue tissues from mice. Notably, among all the OTU family members, *Otud6a* exhibited the most pronounced downregulation of gene expression in the ulcerated tissues (Figure 1A). Correspondingly, OTUD6A protein expression was markedly reduced in ulcerated tissues from mice compared to normal controls (Figure 1B, C). To assess the functional consequence of OTUD6A loss, we generated age‐ and sex‐matched whole‐body *Otud6a* knockout mice (*Otud6a^−/−^*) and *Otud6a^+/+^* littermates. Genotype was confirmed by PCR using tail genomic DNA, which distinguished the wild‐type and knockout alleles (Figure S1A). Successful depletion of OTUD6A protein in mouse tongue tissues was further verified by western blot analysis (Figure S1B, C). As shown in Figure S1D, under untreated conditions, tongue tissues from both *Otud6a^−/−^* mice and their *Otud6a^+/+^* littermates exhibited intact epithelial architecture, normal basement membrane integrity, and no spontaneous inflammatory cell infiltration. Our data showed that *Otud6a* deficiency resulted in significantly larger ulcers, aggravated inflammation, and deeper central depressions (Figure 1D–I). Histopathological examination further revealed that *Otud6a* deficiency markedly exacerbated mucosal damage in mice, characterized by extensive epithelial loss, disruption of the basement membrane, and pronounced inflammatory cell infiltration, reduced collagen deposition, and increased apoptotic activity (Figure 1J–L).

### 2.2 OTUD6A Rescued the Cellular Damage of HOKs

**FIGURE 2:**
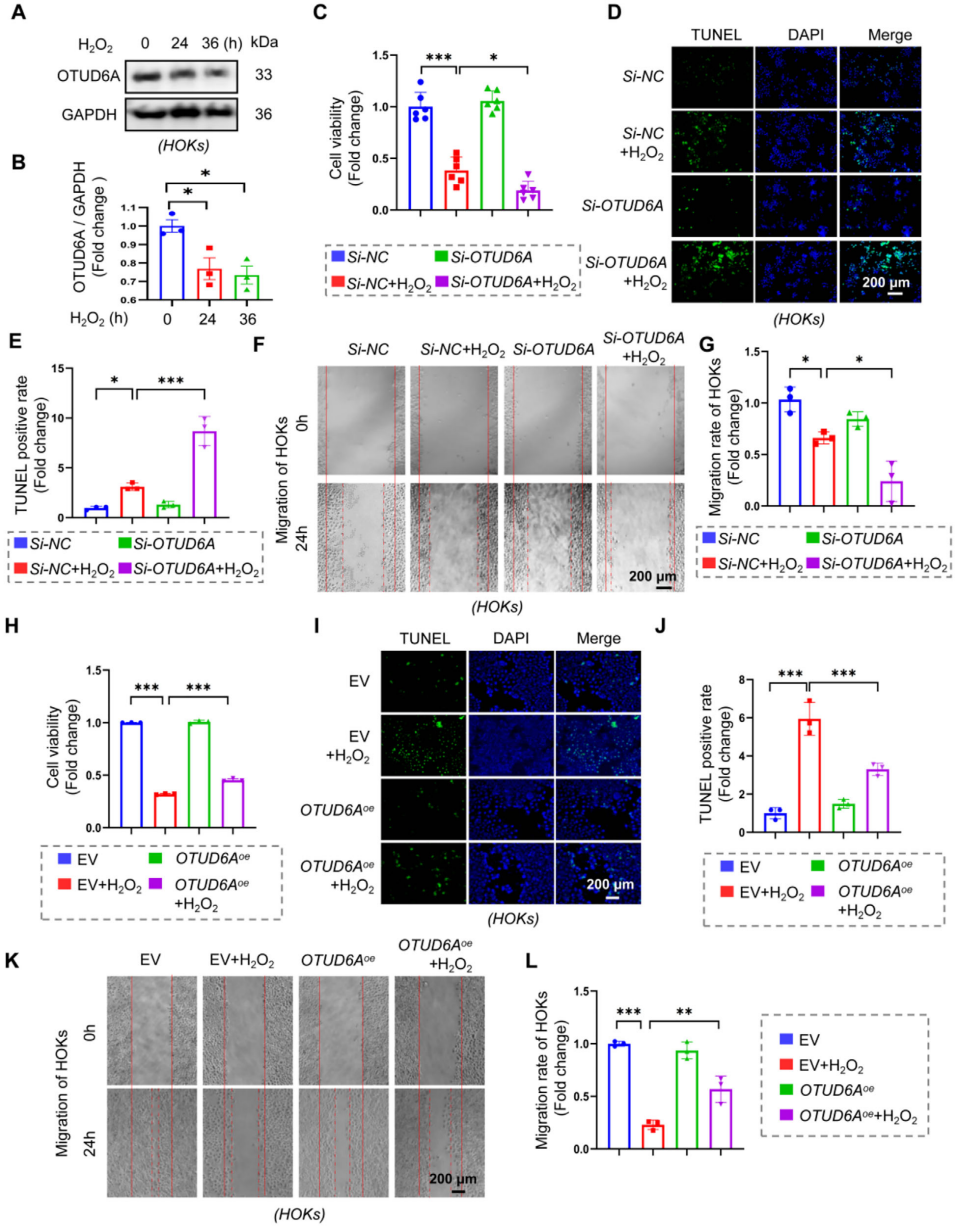
*OTUD6A* overexpression rescued the cellular damage of HOKs. (A, B) Representative quantification and immunoblot of OTUD6A protein in HOKs upon H_2_O_2_ treatment. Data are presented as mean ± SEM, n = 3 per group. (C) Cell viability was quantified using the MTT assay of HOKs transfected with or without *Si‐OTUD6A* (or *Si‐NC*) after H_2_O_2_ exposure. Data are presented as mean ± SEM, n = 6 per group. (D, E) Apoptosis of HOKs was determined by transferase‐mediated dUTP nick end labeling (TUNEL) assay and expressed as the cell apoptotic percentage of HOKs transfected with or without *Si*‐*OTUD6A* (or *Si‐NC*) after H_2_O_2_ exposure. Data are presented as mean ± SEM, n = 3 per group. Scale bar = 200 µm. (F, G) Migration rate of HOKs transfected with or without *Si‐OTUD6A* (or *Si‐NC*) after H_2_O_2_ exposure was analyzed by scratch wound assay. Data are presented as mean ± SEM, n = 3 per group. Scale bar = 200 µm. (H) Cell viability of HOKs transfected with or without *OTUD6A^oe^* (or EV) after H_2_O_2_ exposure was quantified using the MTT assay. Data are presented as mean ± SEM, n = 3 per group. (I, J) Apoptosis of HOKs was determined by transferase‐mediated dUTP nick end labeling (TUNEL) assay and expressed as the apoptotic percentage of HOKs transfected with or without *OTUD6A^oe^* (or EV) after H_2_O_2_ exposure. Data are presented as mean ± SEM, n = 3 per group. Scale bar = 200 µm. (K, L) Migration rate of HOKs transfected with or without *OTUD6A^oe^* (or EV) after H_2_O_2_ exposure was analyzed by scratch wound assay. Data are presented as mean ± SEM, n = 3 per group. Scale bar = 200 µm. ^*^p < 0.05, ^**^p < 0.01, ^***^p < 0.001.

HOKs, a major cellular component of the oral mucosa, play a critical role in ulcer‐related metabolic processes [6, 20]. We established a commonly used hydrogen peroxide (H_2_O_2_)‐induced HOKs damage model to simulate oral mucosal injury [20, 21]. Notably, the protein levels of OTUD6A were downregulated upon H_2_O_2_ stimulation (Figure 2A, B). To further investigate the functional role of OTUD6A, we established HOKs with *OTUD6A* overexpression or knockdown, and subjected them to H_2_O_2_ treatment. The results indicated that H_2_O_2_ exposure markedly decreased cell viability (Figure 2C), enhanced apoptosis (Figure 2D, E), and suppressed cell migration (Figure 2F, G). These detrimental effects were further aggravated by *OTUD6A* knockdown. Conversely, *OTUD6A* overexpression effectively rescued cell viability (Figure 2H), attenuated apoptosis (Figure 2I,J), and enhanced cell migration of HOKs (Figure 2K,L).

### 2.3 OTUD6A Directly Bound to PRDX1 and Deubiquitinated PRDX1 at K136 Residue By Its Active Site C152

**FIGURE 3:**
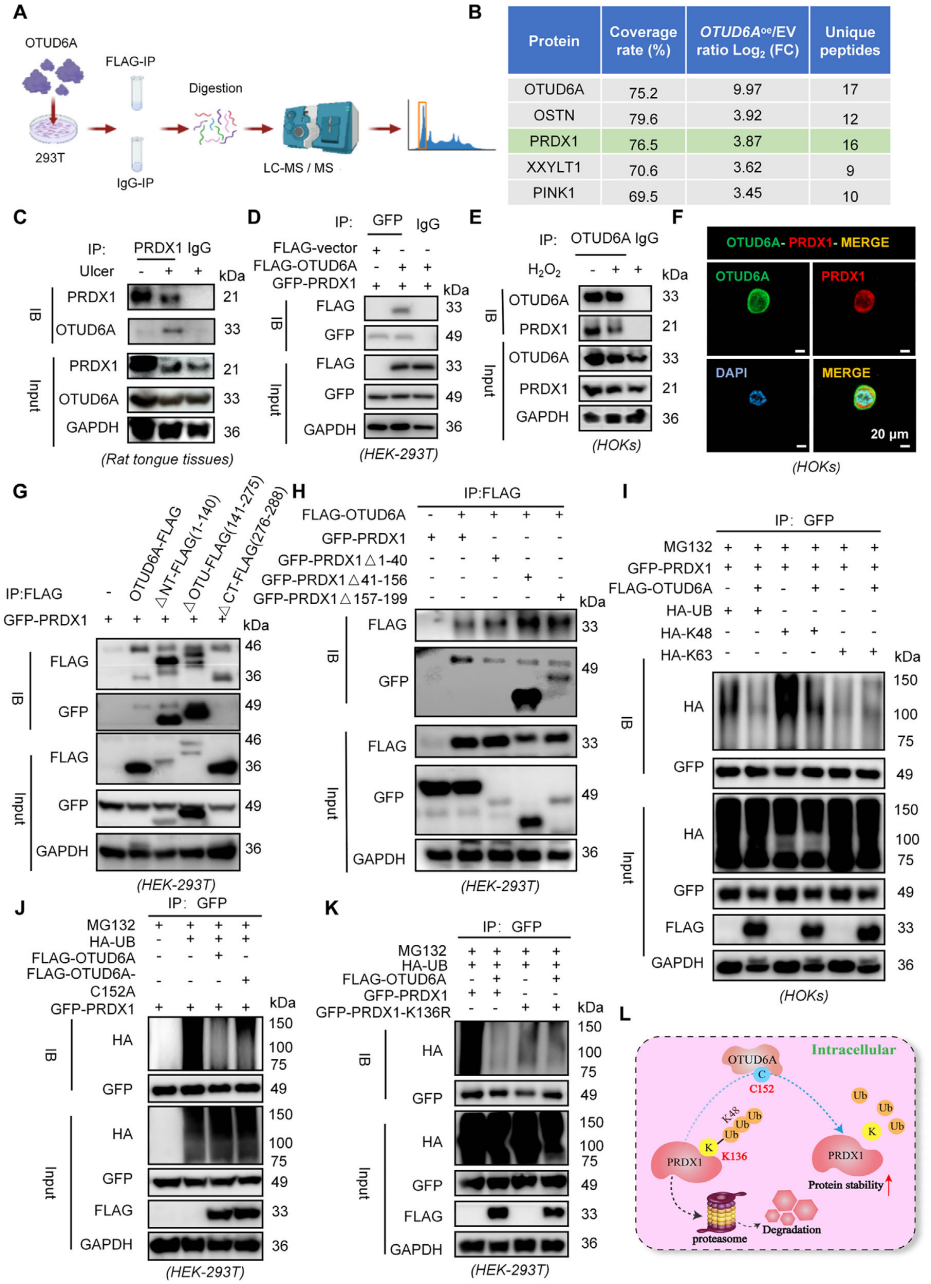
OTUD6A directly bound to PRDX1 and deubiquitinated PRDX1 at K136 residue by its active site C152. (A) Schematic illustration of a quantitative proteomic screen to identify proteins binding to OTUD6A. (B) 4 potential OTUD6A deubiquitination substrates identified from LC‐MS/MS analysis. (C) Co‐immunoprecipitation (Co‐IP) of OTUD6A and PRDX1 in tongue tissue from rat without or with OU, n = 3 per group. (D) Co‐immunoprecipitation (Co‐IP) of GFP‐PRDX1 and FLAG‐OTUD6A in HEK‐293T cells co‐transfected with overexpression plasmids of GFP‐PRDX1 and FLAG‐OTUD6A. OTUD6A immunoprecipitated by anti‐FLAG antibody, n = 3 per group. (E) Co‐immunoprecipitation (Co‐IP) of OTUD6A and PRDX1 in HOKs, n   = 3 per group. (F) Immunofluorescent (IF) staining showing colocalization of OTUD6A and PRDX1 in HOKs, n = 3 per group. Scale bar = 20 µm. (G) FLAG‐tagged OTUD6A or its truncated mutants were transfected into HEK‐293T cells with GFP‐tagged PRDX1. The cell lysates were immunoprecipitated with the anti‐FLAG magnetic beads and then immunoblotted with the indicated antibodies, n = 3 per group. (H) Co‐immunoprecipitation (Co‐IP) was performed using lysates from HEK‐293T cells co‐transfected with FLAG‐OTUD6A and either GFP‐PRDX1‐wt or GFP‐PRDX1‐mut plasmids. Exogenous OTUD6A was immunoprecipitated using an anti‐FLAG antibody, n = 3 per group. (I) Immunoprecipitation of PRDX1 in HOKs cells co‐transfected with GFP‐PRDX1, FLAG‐OTUD6A, and HA‐UB, HA‐UB‐K48 (K48 only), HA‐UB‐K63 (K63 only) and then challenged with 10 µM MG132 for 6 h. Ubiquitinated PRDX1 detected by immunoblotting staining using an anti‐HA antibody, n = 3 per group. (J) Immunoblot analysis of the ubiquitination of PRDX1 in HEK‐293T cells co‐transfected with HA‐UB, GFP‐PRDX1 together with FLAG‐OTUD6A or FLAG‐OTUD6A‐C152A, followed by Co‐IP with anti‐HA magnetic beads. Cells were pretreated with 10 µM MG132 for 6 h before harvesting, n = 3 per group. (K) Immunoprecipitation of PRDX1 in HEK‐293T cells co‐transfected with FLAG‐OTUD6A, HA‐UB, GFP‐PRDX1 and GFP‐PRDX1^K136R^ plasmids. Exogenous ubiquitinated PRDX1 was detected by immunoblotting using a GFP‐specific antibody to identify the active site of OTUD6A that regulate the ubiquitination of PRDX1, n = 3 per group. (L) Scheme for the mechanism of OTUD6A deubiquitinated PRDX1.

DUBs play pivotal roles in diverse biological processes by modulating substrate proteins [22]. To identify potential OTUD6A substrates, we conducted co‐immunoprecipitation (Co‐IP) coupled with liquid chromatography‐tandem mass spectrometry (LC‐MS/MS) for interactome analysis (Figure 3A), which revealed 4 candidate targets. Among them, osteocrin (OSTN) and PRDX1 emerged as a high‐confidence OTUD6A binding partner based on high peptide coverage, high enrichment upon *OTUD6A* overexpression, and number of unique peptides (Figure 3B). OSTN is a small secretory peptide originally cloned from bone tissue and is known as a key regulator of bone metabolism [23]. PRDX1 is an antioxidant enzyme with well‐characterized anti‐inflammatory and anti‐apoptotic properties and has been previously linked to ulcerative colitis [24], prompting us to focus on it for further validation. Notably, the protein expression of PRDX1 was markedly reduced in ulcerated compared to normal tongue tissues in mice (Figure S2A). Subsequent Co‐IP assays further confirmed that OTUD6A directly interacted with PRDX1 in tongue tissues of rats (Figure 3C), HEK‐293T cells expressing exogenous OTUD6A and PRDX1 proteins (Figure 3D), and HOKs (Figure 3E). Their interaction was further supported by immunofluorescence staining showing clear colocalization of OTUD6A and PRDX1 in HOKs (Figure 3F). To further identify the OTUD6A domain essential for PRDX1 binding, we constructed a series of FLAG‐OTUD6A truncation mutants and co‐transfected them with full‐length PRDX1‐GFP vectors into HEK‐293T cells (Figure S2B). Co‐IP analysis revealed that the C‐terminal domain (CTD, residues 276–288) of OTUD6A was required for interaction with PRDX1 (Figure 3G). Furthermore, PRDX1 contains three structural domains: an N‐terminal domain (NTD, aa 1–40), a central domain (aa 41–156), and a C‐terminal domain (aa 157–199) (Figure S2C) [25]. We found that the N‐terminal domain (NTD, aa 1–40) of PRDX1 was identified as essential for its binding to OTUD6A, as demonstrated by Co‐IP assays in HEK‐293T cells (Figure 3H).

To determine whether OTUD6A regulates the deubiquitination of PRDX1, we co‐transfected HEK‐293T cells with plasmids encoding FLAG‐OTUD6A, GFP‐PRDX1, and HA‐UB, followed by proteasome inhibitor MG132 treatment. The results showed that OTUD6A markedly reduced PRDX1 ubiquitination (Figure S2D). Given that K48‐linked ubiquitination typically targets substrates for proteasomal degradation while K63‐linked chains mediate non‐degradative functions [26], we further evaluated the linkage specificity of OTUD6A. Our data indicated that OTUD6A selectively removed K48‐linked polyubiquitin chains from PRDX1, without affecting K63‐linked ubiquitination in HEK‐293T cells (Figure S2E) and HOKs cells (Figure 3I). To assess the dependence on its catalytic activity, we generated a catalytically inactive mutant (OTUD6A‐C152A), as Cys152 within the OTU domain is essential for deubiquitination [27]. As expected, this mutation abolished OTUD6A‐mediated deubiquitination and led to decreased PRDX1 stability (Figure 3J). These results demonstrated that OTUD6A directly deubiquitinated and stabilized PRDX1 by specifically cleaving its K48‐linked ubiquitin chains via its C152 catalytic residue.

Furthermore, using GPS‐Uber prediction, we identified 4 high‐confidence ubiquitination residues: K7, K16, K120, and K136 (Table S1). Systematic lysine‐to‐arginine (K‐to‐R) mutagenesis demonstrated that OTUD6A primarily cleaved ubiquitin chains at the lysine 136 site (K136) of PRDX1 (Figure 3K and Figure S2F–I), establishing K136 as the key site for OTUD6A‐mediated deubiquitination. Collectively, these findings revealed a dual mechanism wherein the NTD (aa 1–40) of PRDX1 facilitated its binding to OTUD6A, and OTUD6A in turn deubiquitinated PRDX1 at K136 via its catalytic residue C152, thereby establishing a novel OTUD6A‐PRDX1 axis that mitigated OU pathogenesis (Figure 3L).

### 2.4 PRDX1 and OTUD6A Attenuated Mitochondrial Oxidative Stress and Dysfunction of HOKs

Our results showed that *PRDX1* overexpression protected HOKs from cellular damage, as reflected by increased viability (Figure S3A), reduced apoptosis (Figure S3B, C), and restored migration capacity (Figure S3D, E). Furthermore, *PRDX1* overexpression alleviated mitochondrial dysfunction enhanced by H_2_O_2_ treatment, as demonstrated by lowering total reactive oxygen species (ROS) and mitochondrial reactive oxygen species (mtROS) levels, and restoring mitochondrial membrane potential (MMP) and adenosine triphosphate (ATP) production (Figure S3F–L). Given the central role of PRDX1 in mitochondrial homeostasis and our finding that OTUD6A stabilized PRDX1, we hypothesized that OTUD6A regulates mitochondrial function through PRDX1. The in vitro results confirmed that *OTUD6A* deficiency exacerbated H_2_O_2_‐induced mitochondrial dysfunction, manifested by increased total and mtROS, decreased mitochondrial membrane potential (MMP), and reduced ATP production (Figure S4A–G). Conversely, *OTUD6A* overexpression alleviated these abnormalities (Figure S4H–N). To determine whether elevated mtROS directly contributes to HOKs dysfunction upon *OTUD6A* loss, we treated *OTUD6A*‐knockdown HOKs with the well‐characterized mitochondrial‐targeted antioxidant Mitoquinone (MitoQ) prior to H_2_O_2_ stimulation [28]. The results confirmed that MitoQ treatment significantly rescued cell viability (Figure S5A), reduced apoptosis (Figure S5B, C), and restored migration in *OTUD6A*‐deficient HOKs (Figure S5D, E). These data confirmed that elevated mtROS contributed to HOKs dysfunction upon *OTUD6A* loss.

Histological analysis of mouse tongue tissues further revealed that *Otud6a* knockout resulted in reduced ATP levels and elevated expression of 8‐hydroxy‐2‐deoxyguanosine (8‐OHDG), the widely used biomarker of oxidative damage (Figure S6A, B).

### 2.5 PRDX1 Abolished the Cellular Damage and Mitochondrial Dysfunction Enhanced by OTUD6A Deficiency

**FIGURE 4:**
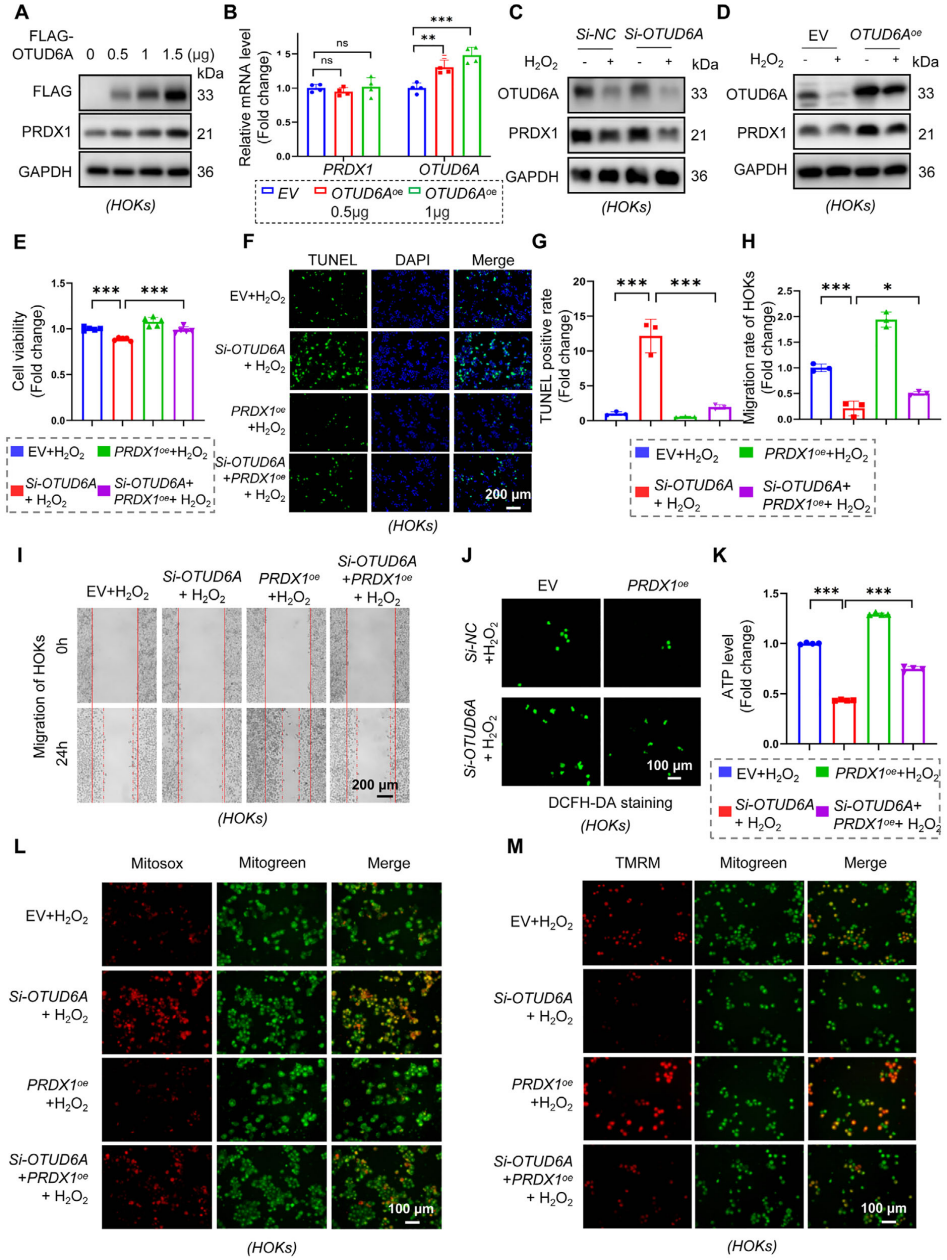
PRDX1 abolished the cellular damage and mitochondrial dysfunction enhanced by *OTUD6A* deficiency. (A) Representative immunoblot of OTUD6A and PRDX1 in HOKs transfected with overexpression plasmids of FLAG‐OTUD6A, n = 3 per group. (B) qRT‐PCR analysis of *OTUD6A* and *PRDX1* in HOKs transfected with overexpression plasmids of FLAG‐OTUD6A, n = 4 per group. (C) Representative immunoblot of PRDX1 and OTUD6A levels in HOKs transfected with *Si‐OTUD6A* after 24 h of H_2_O_2_‐induction, n = 3 per group. (D) Representative immunoblot of PRDX1 and OTUD6A levels in HOKs transfected with overexpression plasmids of FLAG‐OTUD6A after 24 h of H_2_O_2_‐induction, n = 3 per group. (E) MTT assay to quantity cell viability of HOKs transfected with or without *Si‐OTUD6A* (or *PRDX1^oe^*) after H_2_O_2_ exposure. Data are presented as mean ± SEM, n = 5 per group. (F, G) Apoptosis of HOKs transfected with or without *Si‐OTUD6A* (or *PRDX1^oe^*) after H_2_O_2_ exposure was determined by transferase‐mediated dUTP nick end labeling (TUNEL) assay and expressed as the percentage of apoptotic cells. Data are presented as mean ± SEM, n = 3 per group. Scale bar = 200 µm. (H, I) Migration rate of HOKs transfected with or without *Si‐OTUD6A* (or *PRDX1^oe^*) after H_2_O_2_ exposure as analyzed by scratch wound assay. Data are presented as mean ± SEM, n = 3 per group. Scale bar = 200 µm. (J) Representative DCFH‐DA staining images of HOKs transfected with or without *Si‐OTUD6A* (or *PRDX1^oe^*) after H_2_O_2_ exposure, n = 4 per group. Scale bar = 100 µm. (K) ATP content of HOKs transfected with or without *Si‐OTUD6A* (or *PRDX1^oe^*) after H_2_O_2_ exposure. Data are presented as mean ± SEM, n = 4 per group. (L) Representative Mitosox staining images of HOKs transfected with or without *Si‐OTUD6A* (or *PRDX1^oe^*) after H_2_O_2_ exposure, n = 3 per group. Scale bar = 100 µm. (M) Representative TMRM staining images of HOKs transfected with or without *Si‐OTUD6A* (or *PRDX1^oe^*) after H_2_O_2_ exposure, n = 3 per group. Scale bar = 100 µm. Data are presented as mean ± SEM. ^*^p < 0.05, ^**^p < 0.01, ^***^p < 0.001, ns, no significance.

The interaction between OTUD6A and PRDX1 prompted us to investigate whether OTUD6A regulates PRDX1. We found that *OTUD6A* overexpression increased the protein level of PRDX1 (Figure 4A and Figure S7A), without affecting its mRNA level (Figure 4B). Further experiments demonstrated that OTUD6A slowed the degradation of PRDX1 and enhanced its stability (Figure S7B, C). Notably, *Otud6a* knockdown decreased PRDX1 protein levels in tongue tissue from mice (Figure S7D). Moreover, *OTUD6A* knockdown resulted in decreased PRDX1 protein levels upon H_2_O_2_ treatment, while *OTUD6A* overexpression increased PRDX1 protein levels (Figure 4C,D and Figure S7E–H).

We further evaluated whether PRDX1 counteracts the damage caused by OTUD6A loss. The results showed that *OTUD6A* knockdown enhanced cellular injury of HOKs, as evidenced by decreased cell viability, increased apoptosis, and impaired migration (Figure 4E–I). Correspondingly, *OTUD6A* ablation aggravated H_2_O_2_‐induced mitochondrial dysfunction, marked by elevated ROS, increased mtROS, reduced ATP level, and diminished MMP. Importantly, all these adverse effects were rescued by *PRDX1* overexpression (Figure 4J–M and Figure S7I–K).

To determine whether the protective function of OTUD6A depended on PRDX1, HOKs were co‐transfected with *OTUD6A* overexpression vectors and PRDX1‐targeting siRNAs (or *Si‐NC*) followed by H_2_O_2_ treatment, with immunoblotting confirming effective *PRDX1* knockdown (Figure S8A, B). Our data revealed that *PRDX1* knockdown decreased cell viability in *OTUD6A*‐overexpressing HOKs exposed to H_2_O_2_ (Figure S8C), and increased the percentage of Terminal deoxynucleotidyl transferase dUTP Nick end labeling (TUNEL)—positive cells (Figure S8D, E) and suppressed cell migration (Figure S8F, G). Collectively, these results demonstrated that *PRDX1* knockdown largely abolished the protective effects of *OTUD6A* overexpression against H_2_O_2_‐induced injury, confirming that the protective function of OTUD6A was largely dependent on PRDX1.

### 2.6 OTUD6A@HmA Enhanced the Healing of OU in Mice

**FIGURE 5:**
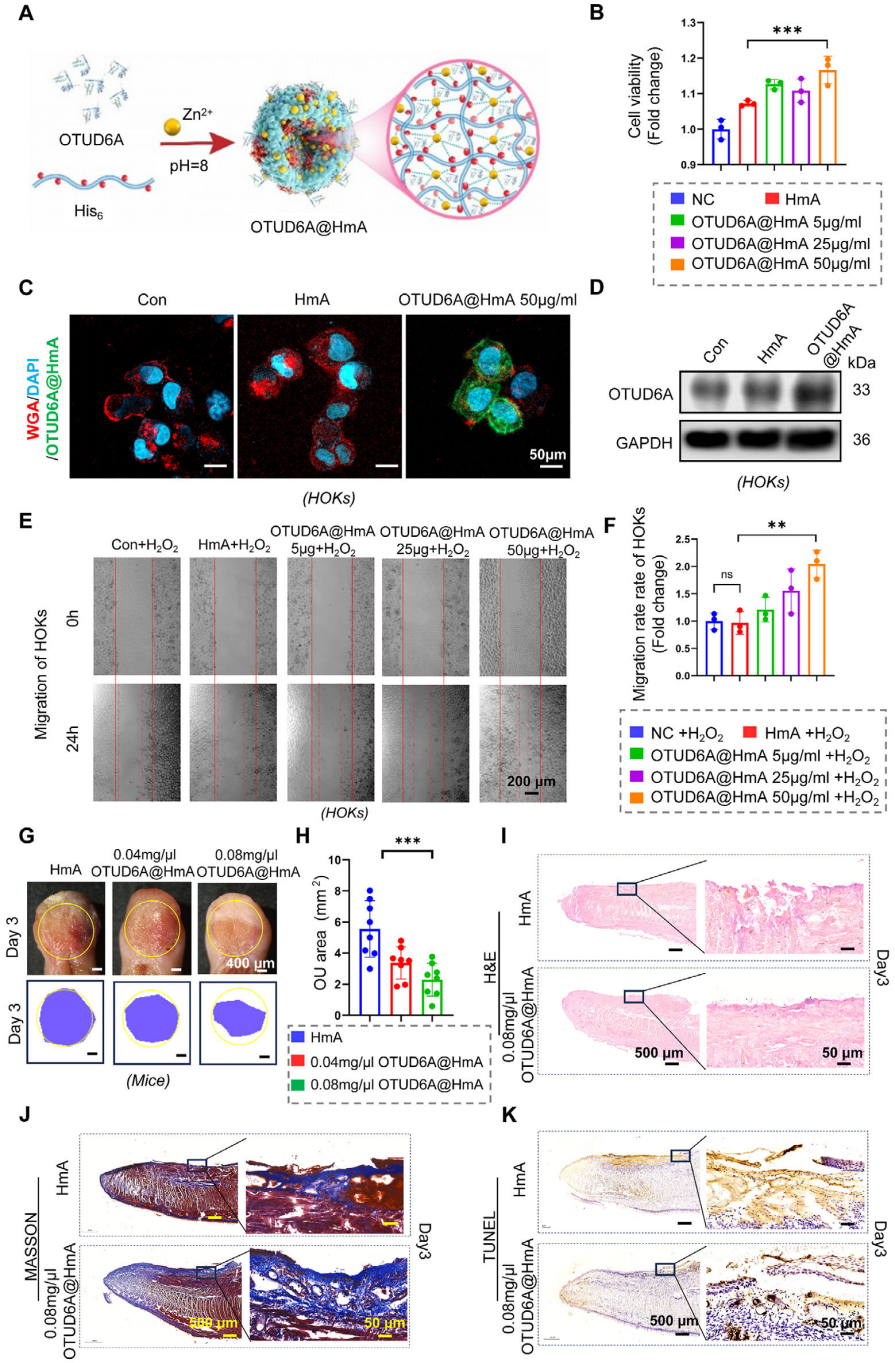
OTUD6A@HmA enhanced the healing of OU in mice. (A) Schematic illustration of the formation of OTUD6A@HmA particles. (B) Cell viability quantified using the MTT assay. Data are presented as mean ± SEM, n = 3 per group. (C) The endocytosis of HmA and OTUD6A@HmA was observed by confocal laser scanning microscopy. Red, blue, and green represent the cell membrane, nucleus, and OTUD6A@HmA, respectively, n = 3 per group. Scale bar = 50 µm. (D) Representative immunoblot of OTUD6A in HOKs cells treated with HmA or OTUD6A@HmA, n = 3 per group. (E, F) Migration rate of HOKs analyzed by scratch wound assay. Data are presented as mean ± SEM, n = 3 per group. Scale bar = 200 µm. (G) Representative images of mouse tongues of the sham group and OTUD6A@HmA‐treated OU of mice on day 3, n = 8 per group. Scale bar = 400 µm. (H) Quantitative assessment of area of OU. Data are presented as mean ± SEM, n = 8 per group. (I) H&E staining of the sham group and OTUD6A@HmA‐treated OU, n = 8 per group. Scale bar = 500 µm (Enlarged image scale bar = 50 µm). (J) Masson staining of the sham group and OTUD6A@HmA‐treated OU, n = 8 per group. Scale bar = 500 µm (Enlarged image scale bar = 50 µm). (K) TUNEL staining of the sham group and OTUD6A@HmA‐treated OU, n = 8 per group. Scale bar = 500 µm (Enlarged image scale bar = 50 µm). ^**^p < 0.01, ^***^p < 0.001, ns, no significance.

Given that *Otud6a* knockout exacerbated ulcer tissue damage in mice, we sought to investigate whether exogenous OTUD6A protein supplementation enhances ulcer healing. Using HmA, a safe and long‐acting protein delivery system previously established by our research group [29], we encapsulated OTUD6A protein into HmA particles (OTUD6A@HmA) and delivered them to HOKs and the ulcerated tissues from *Otud6a^+/+^* mice. Dynamic light scattering confirmed successful formation of OTUD6A@HmA nanoparticles, as indicated by a clear Tyndall effect (Figure 5A and Figure S9A). Scanning electron microscopy (SEM) and transmission electron microscopy (TEM) analysis confirmed that both HmA and its drug‐loaded formulations exhibited a spherical morphology with clearly visible particles. Importantly, encapsulation of OTUD6A proteins did not compromise the morphological integrity of the nanoparticles (Figure S9B, C). The average size of OTUD6A@HmA was 63 ± 3.4 nm, which was larger than empty HmA (44 ± 2.8 nm), further confirming effective encapsulation (Figure S9D). Additionally, the zeta potential decreased from + 33.6 ± 1.6 mV to +24.93 ± 1.2 mV, suggesting OTUD6A incorporation—either within the core or on the surface—while still retaining sufficient positive charge to enhance cellular uptake (Figure S9E). Furthermore, our study confirmed that OTUD6A@HmA nanoparticles exhibited excellent batch‐to‐batch reproducibility, with consistent size and PDI across three independent batches (Figure S9F, G). Moreover, they remained stable in Phosphate‐buffered saline (PBS) for 7 days without significant size change, and only a slight size increase was observed in serum due to protein corona formation (Figure S9H). Furthermore, the PDI remained below 0.25 for 7 days, indicating good dispersion (Figure S9I).

We next determined the encapsulation parameters and release behavior of the nanoparticle formulation. The entrapment efficiency (EE%) of OTUD6A reached 94.4%, as calculated from the standard curve in Figure S9J, with a corresponding drug loading capacity (LC%) of 8.18%. We further evaluated the release profile of OTUD6A@HmA nanoparticles. As shown in Figure S9K, the release of OTUD6A from HmA nanoparticles was markedly pH‐dependent. At physiological pH (7.5), the nanoparticles exhibited minimal release, with a cumulative release rate of approximately 7% over 15 h. Under mildly acidic conditions (pH 6.0), the release was accelerated, reaching a cumulative release of approximately 36% within 3 h and then plateauing with no further significant increase up to 15 h. Notably, under strongly acidic conditions (pH 4.5), a rapid burst release was observed, with the cumulative release reaching approximately 46% within 1 h and exceeding 90% by 3 h, followed by a plateau. These results demonstrated that OTUD6A@HmA nanoparticles possessed excellent pH‐responsive release characteristics, which may facilitate targeted protein delivery to the acidic ulcer microenvironment. The in vitro results further revealed that OTUD6A@HmA increased the viability of HOKs (Figure 5B) and improved the cellular uptake of OTUD6A protein (Figure 5C,D). Scratch assays showed that OTUD6A@HmA treatment promoted HOKs migration in a dose‐dependent manner, while HmA alone had no effect (Figure 5E, F). Moreover, the in vivo results demonstrated that OTUD6A@HmA enhanced OU healing in mice in a dose‐dependent manner, as evidenced by reduced ulcer area and depth, and less weight loss (Figure 5G,H, and Figure S9L–N). Histological analysis further demonstrated that on day 3 of treatment, the OTUD6A@HmA group exhibited accelerated ulcer healing, characterized by attenuated inflammation, enhanced subepithelial cell proliferation, and increased collagen deposition (Figure 5I,J). In contrast, ulcers without supplementation of OTUD6A protein presented persistent inflammation, accompanied by elevated apoptosis and delayed re‐epithelialization (Figure 5K). Consistently, OTUD6A@HmA treatment upregulated the expression of both OTUD6A and PRDX1 in the tongue tissues of mice (Figure S9O–P).

## 3 Discussion

As one of the most common oral mucosal diseases, OU remains therapeutically challenging due to its high recurrence rate and considerable clinical morbidity. Current clinical management relies primarily on topical anesthetics and analgesics for symptomatic relief, with corticosteroids used in severe or refractory cases [30, 31]. A deeper and more comprehensive understanding of OU will facilitate the development of innovative therapeutic strategies. Our study demonstrated that a DUB, OTUD6A, played a protective role in OU. Deficiency of OTUD6A exacerbated ulcer severity, whereas administration of OTUD6A protein via HmA nanoparticles (OTUD6A@HmA) significantly promoted OU healing in mice. Mechanistically, OTUD6A directly bound to the N‐terminal domain (residues 1–40) of PRDX1 and specifically cleaved K48‐linked polyubiquitin chains at K136 via the catalytic residue C152, thereby stabilizing PRDX1 in HOKs. Loss of OTUD6A led to PRDX1 degradation, which enhanced mitochondrial oxidative stress and dysfunction, ultimately impairing the function of HOKs. Correspondingly, *OTUD6A* overexpression enhanced the proliferation and migration of HOKs and suppressed apoptosis. Collectively, our results identified OTUD6A as a key regulator in OU pathogenesis and suggested its potential as a therapeutic target for OU treatment.

Ubiquitination, a reversible post‐translational modification, regulates protein degradation and function and is essential in cellular signaling, inflammation, immunity, and disease. DUBs counteract this process by removing ubiquitin chains to stabilize substrate proteins. Despite the identification of nearly 100 DUBs in humans, research on their roles in oral diseases remains limited [12]. Especially, no specific DUB has been reported to be involved in OU pathogenesis so far. Our study identified OTUD6A as a novel protective agent against OU, marking the first report of a functional role for DUBs in OU pathology. This finding aligns with the growing interest in targeting DUB family members for the treatment of OU.

DUBs regulated protein stability by removing ubiquitin chains from substrate proteins [32]. Here, we identified PRDX1 as a potential substrate of OTUD6A. PRDX1 is a 2‐Cys antioxidant enzyme that scavenges ROS to maintain intracellular redox balance, thereby alleviating oxidative stress and mitochondrial dysfunction [33, 34, 35]. Mounting evidence indicates that excessive ROS production contributed to refractory wound pathologies, including diabetic foot ulcers and ulcerative colitis, by inducing mitochondrial dysfunction [36, 37]. Notably, while increased PRDX1 expression has been linked to the progression of ulcerative colitis [38], its role in OU remains elusive [39, 40]. Our study revealed that PRDX1 deletion enhanced oxidative damage and mitochondrial dysfunction, accelerating the disruption of HOKs and exacerbated OU progression. Critically, *PRDX1* overexpression reversed the oxidative damage and mitochondrial dysfunction enhanced by *OTUD6A* deficiency, establishing the OTUD6A–PRDX1 axis as a key pathway in maintaining mitochondrial homeostasis. Moreover, despite the established mucosal protective roles of PRDX1, no specific small‐molecule activators of PRDX1 are available so far. Our results demonstrated that targeted delivery of OTUD6A protein alleviated OU by enhancing PRDX1 stability and protein level. Consequently, developing OTUD6A‐targeted agents represents a specific and viable therapeutic strategy to therapeutically upregulate PRDX1.

Although PRDX1 was initially regarded as a predominantly cytoplasmic protein, recent microscopic studies have revealed its presence in multiple subcellular compartments, including the nucleus, mitochondria, and peroxisomes [33, 34, 35]. PRDX1 has been known to be regulated at transcriptional levels in cells [41], but also through a variety of post‐translational modifications, including ubiquitination, acetylation, phosphorylation, glutathionylation, and S‐nitrosylation [33, 34, 35]. While E3 Ligase TRIM16 and DUBs such as OTUD1 have been shown to regulate PRDX1 ubiquitination, the full scope of the regulatory landscape of PRDX1 remains unclear [42, 43]. In this study, we identified a new PRDX1‐regulating DUB and meticulously delineated the mechanism by which OTUD6A stabilized PRDX1 by binding to the N‐terminal domain of PRDX1 to cleave K48‐linked ubiquitin chains at the K136 residue of PRDX1.

Despite the established protective role of OTUD6A in OU, no small‐molecule activators have been reported — a gap that reflects the general challenge in developing DUB agonists. While most efforts in the DUB field have focused on inhibitors, such as those targeting USP7 for cancer therapy [44], emerging technologies like deubiquitinase‐targeting chimeras offer new avenues for pharmacological DUB activation [45, 46]. Future development of OTUD6A‐specific agonists may provide a targeted therapy for OU. However, the challenge of efficiently delivering the OTUD6A protein itself, a prerequisite for clinical efficacy, must be overcome to advance clinical application. Building on our previous work [29], we encapsulated OTUD6A protein using HmA nanoparticles—a safe, long‐acting delivery system with high encapsulation efficiency, positive surface charge, pH‐responsive release, and good biocompatibility [47]—a platform our group has validated for insulin release [29] and vaccine delivery [48]. Our systematic in vitro and in vivo studies demonstrated that OTUD6A@HmA significantly accelerated OU healing. These results not only highlight the promise of HmA nanoparticles for delivering therapeutic deubiquitinases, but also provide a strong foundation for clinical translation, opening new avenues for next‐generation OU therapeutics. Current clinical strategies for OU, such as commercial patches containing flavonoids and chitosan and growth factor‐based gel ointments, are often limited by poor mucosal adhesion, short intraoral retention time, and low drug efficacy in the wet and dynamic oral environment [49]. Therefore, the development of a robust mucoadhesive protein delivery system is essential to improve OU treatment. Our OTUD6A‐loaded HmA nanoparticle platform has the potential to overcome these limitations, thereby offering a promising and translationally enhanced therapeutic strategy.

While this study provides valuable insights, it has some limitations. Although the role of OTUD6A in HOKs is established, the use of oral keratinocyte‐specific *Otud6a^−/−^* mice would further substantiate its function and underlying mechanism in these cells. Furthermore, the function of OTUD6A in other key oral mucosal cell types, particularly in immune cells such as macrophages and neutrophils, which are central to OU pathogenesis, remains unclear [8]. Moreover, given that DUBs often have multiple substrates, we cannot exclude the possibility that OTUD6A may regulate other signaling pathways in OU, such as STAT3, which is a well‐established contributor to OU pathogenesis and has been previously linked to OTUD6A activity in other cellular contexts [50, 51]. While the H_2_O_2_ model is widely used to simulate oral mucosal injury, it does not fully capture the complex OU microenvironment—which includes bacterial components, inflammatory cells, and multiple cytokines [50, 51, 52]. Thus, our findings may not fully recapitulate the multifactorial nature of OU pathology. Future validation using LPS+IFNγ stimulation or immune co‐cultures is warranted. Finally, clinical studies are warranted to evaluate whether supplementation of OTUD6A protein alleviates symptoms in patients with OU.

## 4 Conclusion

Our study established the OTUD6A–PRDX1–mitochondrial dysfunction axis as essential for HOKs homeostasis in OU pathology, showing for the first time that OTUD6A promoted migration and inhibited apoptosis of HOKs by deubiquitinating and stabilizing PRDX1, thereby positioning OTUD6A as a promising therapeutic target for OU.

## 5 Experimental Section/Methods

### 5.1 Animal Study

All the animal experiments were conducted in accordance with the ARRIVE 2.0 guidelines. All the animal care and experimental procedures were approved by the Animal Policy and Welfare Committee of Wenzhou Medical University (approval No. xmsq 2024‐0142). All the animal studies were performed in accordance with the Guide for the Care and Use of Laboratory Animals (National Institutes of Health, USA). 8‐week‐old C57BL/6 wild‐tdataype (*Otud6a^+/^* ^+^) and OTUD6A knockout (*Otud6a^−/−^*) mice on a C57BL/6 background were obtained from Gempharmatech (Nanjing, China) using CRISPR‐Cas9 technology. Briefly, guide RNAs (gRNAs) targeting exon 2 of the mouse *Otud6a* gene were designed to induce frameshift mutations. Cas9 mRNA and gRNAs were co‐injected into fertilized C57BL/6 embryos. Founder mice were genotyped by PCR and Sanger sequencing to confirm the presence of frameshift mutations. Heterozygous offspring were then intercrossed to generate homozygous *Otud6a^−/−^* mice and their wild‐type littermates. Genotyping was performed by PCR using tail genomic DNA. Homozygous knockout was confirmed by the absence of the wild‐type band (516 bp) and the presence of the mutant band (∼350 bp for *Otud6a^−/−^*). The mice were randomly allocated into experimental and control groups. All the mice were housed under specific pathogen‐free conditions with 50 ± 5% humidity at 22 ± 2°C with a 12/12 h light/dark cycle and fed standard rodent chow. Animals were acclimatized to the laboratory for at least 2 weeks before the start of the studies. All the animal experiments were performed and analyzed by blinded experimenters. The exact number of experimental units allocated to each group was determined by a priori power calculation using G‐Power 3.1.9 (http://www.gpower.hhu.de/), with a statistical power of 80% and α = 0.05. and the 3R principles (Replacement, Reduction, and Refinement) [53]. No animals or data points were excluded from the study. All the animals that underwent ulcer induction were included in the final analyses. No predefined exclusion criteria were applied. The exact number of animals used in each experiment is indicated in the figure legends.

### 5.2 Animal Experiment Grouping

To elucidate the role of OTUD6A in OU pathogenesis and treatment, we first established OU models in both OTUD6A knockout mice (*Otud6a^−/−^*) and wild‐type (*Otud6a^+/+^*) littermates, systematically comparing ulcer progression at critical timepoints. Building on these genetic findings, we then employed HmA nanoplatform to deliver recombinant OTUD6A (OTUD6A@HmA) vs. vehicle control (HmA) in *Otud6a^+/+^* mice with OU, enabling comprehensive therapeutic evaluation of this targeted protein delivery system. Animals were housed in identical cages and handled in the same order each day to minimize potential confounders. Experimental procedures and outcome assessments were performed at consistent times of day to reduce circadian influences.

### 5.3 Establishment of OU Model

OU induced by acid etching was performed on the mouse tongue, as previously described [54]. Briefly, mice were anaesthetized with a combination of pentobarbital (50 mg/kg) by intraperitoneal injection. For OU induction, a piece of 3 mm diameter filter paper was soaked in 80% acetic acid and placed on the ventral mucosa of the tongue for 2 mins [6, 8]. The mice were allowed to recover on a controlled heating pad and were maintained on a high‐viscosity liquid diet. OU was formed within 24 h post‐induction.

### 5.4 Pain Assay

Pain assessment was conducted 24 h after model induction (Day 2) in a quiet room. A test box measuring 30×30×20 cm with a grid floor was used. Each animal was acclimated in the box for 15 min to reduce stress, followed by recording of grooming behaviors. The recording was split into three 3‐min blocks. Pain levels were assessed based on the number of facial wiping episodes and the duration of mouth rubbing (or licking) per block. Food and water were withheld during testing. Behavioral analysis was carried out by an investigator blinded to group allocation.

### 5.5 Synthesis and Characterization of OTUD6A@HmA Nanoparticles

The OTUD6A@HmA nanoparticles were fabricated according to previous reports [55]. Briefly, 281 µL of 100 mM 4‐(2‐hydroxyethyl)‐1‐piperazineethanesulfonic acid (HEPES) buffer (pH 8.5) was mixed with 200 µL ddH_2_O and 53 µL hexahistidine solution (40 mg/mL), followed by dropwise addition of 25.7 µL 0.1 M Zn(NO_3_)_2_·6H_2_O under ultrasonication (300 W, 80 kHz) [56]. The characteristic color transition to light blue indicated successful HmA formation. For OTUD6A encapsulation, 200 µL of OTUD6A protein solution (1 mg/mL) was incorporated before 10‐min sonication and subsequent centrifugation (8,000×g, 10 min). The pelleted nanoparticles were resuspended and stored at 4°C.

Dynamic light scattering (DLS) analysis was used to reveal the size, polydispersity index (PDI) and zeta potential of HmA and OTUD6A@HmA [55]. To evaluate batch‐to‐batch reproducibility, three independent batches of HmA and OTUD6A@HmA nanoparticles were prepared under identical conditions. For each batch, the size and PDI were measured using DLS. Moreover, the storage stability of OTUD6A@HmA nanoparticles was assessed by incubating the nanoparticles in PBS (pH 7.4) or fetal bovine serum (FBS) at 37°C for up to 7 days. At predetermined time points (day 1, day 3, and day 7), aliquots were collected, and the hydrodynamic size and PDI were measured by DLS. Each experiment was performed in triplicate. Furthermore, the morphology of HmA and OTUD6A@HmA nanoparticles was examined using SEM (SU8010, Hitachi, Japan) and TEM (FEI Talos F200S, USA) following established protocols [29, 56]. For SEM, the nanoparticle suspension was dropped onto a silicon wafer, air‐dried, and sputter‐coated with platinum for 60 s. Images were captured at an accelerating voltage of 5 kV [56]. For TEM, a drop of the sample was placed onto a carbon‐coated copper grid and dried at room temperature. The samples were then imaged at an accelerating voltage of 80 kV [29].

### 5.6 Encapsulation and Release

Protein encapsulation efficiency (EE%), and drug loading capacity (LC%) were determined, according to the previously well‐established protocol [56].The concentrations of OTUD6A in supernatant or the release medium were measured by ultraviolet spectrophotometry, as calculated from the standard curve (Figure S9J) using the absorbance at 280 nm. The release of OTUD6A from OTUD6A@HmA was tested according to previous reports [57, 58], with slight modification. In brief, OTUD6A@HmA nanoparticles containing 100 µg of OTUD6A protein was set into a dialysis bag with maximum cutting‐off molecular weight 100 kDa, which was then immersed in a 5 mL centrifuge tube containing release medium PBS at different pH conditions (pH 7.5, pH 6.0, and pH 4.5). At predetermined time points, 10 µL of the release medium was collected and measured using a UV–vis spectrophotometer, then an equal volume of fresh release medium was replenished after each sampling.

### 5.7 In Vivo Application of OTUD6A@HmA

For in vivo evaluation, *Otud6a^+/+^* mice were randomly allocated to receive either vehicle control (0 mg/µL) or OTUD6A@HmA (0.04 and 0.08 mg/µL) via daily topical application for 3 consecutive days, commencing 24 h post‐ulcer induction. Animals were monitored twice daily and humanely sacrificed 72 h post‐induction (48 h after final treatment).

### 5.8 Macroscopic Evaluation

Mice were monitored daily for body weight changes. Ulcer diameter and depth were measured at 48‐ and 120‐h post‐induction using digital calipers. Clinical inflammation was evaluated in a single‐blind manner and graded on a scale of 0 to 3 [6].

### 5.9 Histological Evaluation

OU tissues underwent 24‐h fixation in 4% formaldehyde, followed by standard ethanol dehydration and paraffin embedding. Serial 4‐µm sections were obtained from wound margins for histological processing. Tissue sections were first subjected to Hematoxylin and Eosin (H&E) staining (Beyotime, China, and then TUNEL staining was performed to visualize the cell apoptosis rate (Roche, USA), involving PBS washes, Triton X‐100 permeabilization, and specific staining procedures. Parallel Masson trichrome staining (Polysciences, USA) enabled clear differentiation of collagen fibers (blue) from muscle fibers (red), providing a quantitative assessment of both collagen depletion and ulcer severity [59].

### 5.10 Immunohistochemistry and Immunofluorescence Staining

Immunohistochemical analysis was performed on 4‐µm paraffin‐embedded mouse OU sections to evaluate PRDX1 and OTUD6A expression. Tissue sections were incubated overnight at 4°C with rabbit monoclonal anti‐PRDX1 (Cell Signaling Technology, Cat# D5G12) and anti‐OTUD6A antibodies (Proteintech, Cat# 24486‐1‐AP) diluted 1:200 in background‐reducing diluent. Following primary antibody incubation, sections were treated with appropriate secondary antibodies at room temperature. Stained sections were digitally imaged using a high‐resolution slide scanning system. For immunofluorescence staining, HOKs were cultured on 24‐well plates and treated with fluorescently labeled OTUD6A@HmA nanoparticles to assess cellular uptake efficiency. Following 24‐h incubation, cells were fixed with 4% paraformaldehyde (PFA), permeabilized with 0.2% Triton X‐100, and stained with phalloidin‐AlexaFluor 555 and 4',6‐diamidino‐2‐phenylindole (DAPI) for cytoskeletal and nuclear visualization. All the samples were analyzed using high‐resolution slide scanning and confocal microscopy (Nikon A1R system) to determine the subcellular distribution of OTUD6A and its interaction partners.

### 5.11 Cell Lines and Treatment

HOKs were obtained from the American Type Culture Collection. HEK‐293T cells (Cat# GNM 6) were purchased from the Shanghai Institute of Biochemistry and Cell Biology. HOKs and HEK‐293T cells were cultured in Dulbecco's modified eagle medium (DMEM) containing 10% FBS, 100 U/ml streptomycin, and 100 U/ml penicillin. For in vitro simulation of oral mucosal injury, HOKs were treated with 100 µM of H_2_O_2_ (Sigma‐Aldrich, Cat# H1009) for 24 h [20, 21]. To investigate whether elevated mtROS directly contributes to HOKs dysfunction upon OTUD6A deficiency, *OTUD6A*‐knockdown HOKs were treated with the mitochondrial‐targeted antioxidant MitoQ prior to H_2_O_2_ stimulation. Briefly, HOKs were transfected with *Si‐OTUD6A* or *Si‐NC* for 24 h, followed by incubation with MitoQ for 2 h. Cells were then exposed to 100 µM of H_2_O_2_ for an additional 24 h. Subsequently, cell viability, apoptosis, and migration rate were evaluated. Each experiment was performed in triplicate.

### 5.12 Gene Knockdown and Overexpression

Gene silencing and overexpression were achieved by transfecting cells with specific small interfering RNAs (siRNAs) and plasmids, respectively. The siRNA targeting OTUD6A was used with the following sequences: 5'‐GUCUGGAGAUGAAAGCGAUTT‐3' and 5'‐AUCGCUUUCAUCUCCAGACTT‐3'. For PRDX1 silencing, the siRNA sequences were as the following sequences: 5'‐GAUGAGACUUUGAGACUAGUTT‐3' and 5'‐AACUAGUCUCAAAGUCUCAUCTT‐3'. Various expression plasmids, including FLAG‐OTUD6A (wild‐type and the C152A mutant), GFP‐PRDX1 (wild‐type, deletion mutants Δ1‐40, Δ41‐156, Δ157‐199, and lysine mutants K7R, K16R, K120R, K136R), as well as HA‐UB, HA‐K48, and HA‐K63, were constructed by Genechem. Transfections of human oral keratinocytes (HOKs) and HEK‐293T cells with siRNAs or plasmids were performed using Lipofectamine 3000 (Thermo Fisher Scientific, USA).

### 5.13 3‐(4,5‐Dimethylthiazol‐2‐yl)‐2,5‐Diphenyltetrazolium Bromide (MTT) Assay

HOKs were seeded (5 × 10^3^ cells/well) into a 96‐well plate. Following treatment with H_2_O_2_, 20 µl MTT reagent (5 mg/ml; Beijing Solarbio Science & Technology Co., Ltd.) was added to each well for 4 h at 37°C. Subsequently, 150 µl Dimethyl sulfoxide (DMSO) was used to dissolve the purple formazan. Absorbance was measured at a wavelength of 570 nm using a microplate reader (Bio‐Rad Laboratories, inc.).

### 5.14 Scratch‐Test Assay (“Wound Healing Assay”)

Cells were seeded in a 12‐well plate (2×10^5^ cells/well) and treated with different conditions. After 24 h, a sterile 200 µl Pipette‐Tip was used to make a straight scratch line on the monolayer of confluent cells at the bottom of the culture plate. The debris was washed away with PBS, and then the cells were treated with H_2_O_2_ as described above. The cells were then cultured at 37°C, humidified 5% CO_2_. At time point 0 and 24 h, the “wound” areas were observed and recorded by an inverted microscope at the same position of the culture plate. The area of “wound healing” was analyzed using the NIH ImageJ software.

### 5.15 TUNEL Staining

TUNEL staining was conducted using Dead End Colorimetric TUNEL System (Promega Corporation, Fitchburg, WI, USA) according to the manufacturer's instructions. TUNEL‐positive cells were calculated from at least 3 randomly selected fields (10×) per slide.

### 5.16 Determination of Intracellular ROS and Mitochondrial Imaging Assays

HOKs were cultured in 96‐well plates and stimulated under different conditions. Cells were then incubated with 2',7'‐Dichlorodihydrofluorescein Diacetate (DCFH‐DA, 1:1,000, Beyotime, China, Cat# S0033S) for 30 min at 37°C and washed three times with PBS.

The cells were then incubated with tetramethylrhodamine methyl Ester (TMRM) probes (1 µM,Thermo Fisher Scientific, USA, Cat# I34361) and Mitogreen probes (1 µM, Thermo Fisher Scientific, USA, Cat# M7514) to detect mitochondrial membrane potential (MMP) and Mitosox probes (1 µM, Thermo Fisher Scientific, USA, Cat# M36008) and Mitogreen probes for detection of mtROS levels. Images were captured using a fluorescence microscope (Zeiss) and quantified using NIH ImageJ software.

### 5.17 ATP Evaluation

HOKs were cultured in 96‐well plates and stimulated under different conditions. Whole‐cell lysates were prepared using lysis buffer from an ATP assay kit (Beyotime, China). After centrifugation, the supernatants were collected. Luminescence was measured using a luminometer and ATP detection buffer.

### 5.18 RNA Extraction and qRT‐PCR

qRT‐PCR: RNA was extracted with Trizol (Invitrogen, Cat# 15596026) and reverse‐transcribed. qRT‐PCR was performed using SYBR Green Master Mix (Vazyme, China) on a LightCycler system (Roche, Germany). The primers of targeted genes are listed in Table S2, β‐actin served as an internal control.

### 5.19 Proteomics Analysis and LC‐MS/MS

Protein extraction and trypsin digestion. Cell samples were lysed in cold lysis buffer (8 M urea and 1% protease inhibitor cocktail). The supernatant was collected, and the protein concentration was determined. The protein solution (200 µg for each sample) was reduced with dithiothreitol (5 mM, 56°C, 30 min) and alkylated with iodoacetamide (11 mM, room temperature, 15 min) in the dark. The protein solution was first digested with trypsin (1:50 trypsin to protein mass ratio, 37°C, overnight) and then digested with trypsin again (1:100 trypsin to protein mass ratio, 37°C, 4 h).

Ubiquitinated peptide enrichment and LC‐MS/MS. The peptides obtained by digestion were used for ubiquitin peptide enrichment (95%) and protein quantification (5%). The peptides used for ubiquitin peptide enrichment were dissolved in NaCl‐EDTA‐Tris‐Nonidet P‐40 buffer and incubated with anti‐diglycine remnant (K‐ε‐GG) pan antibody beads (CST and PTM Biolabs; 4°C, overnight) with gentle shaking. LC‐MS/MS and subsequent combined ubiquitinome, proteome, and interactome analyses were performed by PTM Biolabs. Based on mass spectrometry data, we identified potential OTUD6A substrate proteins by evaluating their peptide coverage rate, the enrichment upon *OTUD6A* overexpression, and the number of unique peptides. The TOP 20 OTUD6A‐interacting proteins identified by LC‐MS/MS are listed in Table S3.

### 5.20 Western Blotting and Co‐IP

Total protein from cells was extracted using lysis buffer (Boster Biological Technology, USA, Cat#AR0103). Proteins were separated using 10% sodium dodecyl sulfate polyacrylamide gel electrophoresis (SDS–PAGE) and transferred to polyvinylidene fluoride membranes. Before adding specific primary antibodies, membranes were blocked with 5% skim milk for 1.5 h at room temperature. Then, the membranes were incubated overnight at 4°C with the primary antibodies as follows: OTUD6A (1:1000), PRDX1 (1:1000), HA (1:1000), GFP (1:1000), Flag (1:1000) and GAPDH (1:1000). Protein bands were detected by incubation with horseradish peroxidase‐conjugated secondary antibodies and an enhanced chemiluminescence reagent (Bio‐Rad, Hercules, CA, USA). Band densities were quantified using Image J software and normalized to loading controls.

For Co‐IP assays, cell extracts prepared following the treatments were incubated with the indicated antibodies at 4°C overnight. Then the proteins were immunoprecipitated with Protein A+G Agarose (Beyotime, China, Cat# P2012) at 4°C for 4 h. Immunoprecipitation samples were immunoblotted for coprecipitated protein detection. Total lysates were subjected to western blotting analysis as input controls. Protein interactions were quantified using ImageJ software. For ubiquitination assays, the procedure was modified to disrupt non‐covalent interactions. After treatment with 10 µM MG132 for 6 h, cells were lysed in Radioimmunoprecipitation Assay buffer (RIPA) buffer containing protease inhibitors. The lysates were then boiled in the presence of 1% SDS for 10 min, followed by a 10‐fold dilution with RIPA buffer without SDS. The diluted lysates were immunoprecipitated with specific antibodies as described above.

#### 5.20.1 Cycloheximide (CHX) Chase Assay

HEK‐293T cells were transfected with FLAG‐OTUD6A plasmid for 24 h. Then, cells were treated with CHX (25 µg/mL; MedChemExpress, Cat# HY‐12320) for 0, 4, or 8 h. At each time point, cells were collected and lysed. Protein expression levels of OTUD6A (detected by FLAG antibody) and PRDX1 were analyzed by Western blot. GAPDH was used as a loading control. Protein band intensities were quantified using ImageJ, and the degradation rate of PRDX1 was calculated.

### 5.21 Immunofluorescence Staining

HOKs cultivated on slides were subjected to different treatments. The cells were fixed with 4% PFA for 10 min and permeabilized with 0.1% Triton X‐100 for 5 min at room temperature. Next, the cells were blocked with 5% bovine serum albumin for 30 min at room temperature and incubated with anti‐OTUD6A (1:600, Biorbyt, UK, Cat# orb185712) and anti‐PRDX1 (1:200, Abcam, USA, Cat# ab109498) antibodies overnight at 4°C, followed by the corresponding secondary antibody for 1 h at 37°C. The slides were mounted with DAPI‐containing medium and coverslipped for fluorescence microscopy. Nikon Eclipse Ti‐U Microscope (Nikon, Japan) with NlS‐Elements software (y4.5000.1117.0) and Zeiss Axio Scope A1 Microscope (ZEISS, German) were used to acquire images.

### 5.22 Statistical Analysis

All the experiments were randomized and blinded. All the data are expressed as mean±SEM Statistical analyses were performed using GraphPad Prism 8.0 software (GraphPad, San Diego, CA, USA). Comparisons between two groups were analyzed using Student's t‐test. For comparisons involving more than two groups, one‐way ANOVA was used as appropriate. For experiments comparing multiple treatment groups to a single control group, Dunnett's post hoc test was applied. For experiments requiring full pairwise comparisons among all the groups, Tukey's post hoc test for multiple comparisons was used. A p‐value < 0.05 was considered statistically significant. Post hoc tests were performed only when the overall ANOVA F‐test reached p < 0.05, and there was no significant violation of homogeneity of variance.

## Author Contributions

Xiaoyu S., Ruiwei J., and Xingbei P. contributed to conceptualization, data curation, formal analysis and Writing – original draft. Jun M., Lei Y., Yuanyuan C., and Xinyu H. contributed to data curation, formal analysis, funding acquisition and investigation. Yifan P., R Huang., Jiahong C., Ziyi Y., Hongjieliang W., and Yilin M. contributed to methodology, project administration, resource software, and visualization. Huining W., Shengbin H., and Guang L. contributed to conceptualization, validation, Writing – review & editing, and supervision of the manuscript, and final approval. All authors agree to be accountable for the integrity and accuracy of the work.

## Funding

The authors disclosed receipt of the following financial support for the research: This study was supported by the National Natural Science Foundation of China (82571103, 82270991 and 82571101); Zhejiang Provincial Key Scientific Project (LY21H140003); Zhejiang Provincial Medical and Health Science and Technology Plan Project (2024KY164), and the Foundation for the new star of the medical industry in Zhejiang Province. Medical Health Science and Technology Major Project of Zhejiang Provincial Health Commission (WKJ‐ZJ‐2311). The Summit Advancement Disciplines of Zhejiang Province (Wenzhou Medical University – Pharmaceutics). The Provincial Advantageous Characteristic Discipline of Wenzhou Medical University (Clinical Medicine). The Wenzhou Municipal Science and Technology Bureau of China (Y20240329). The authors thank Scientific Research Center of Wenzhou Medical University for providing excellent consultation and instrumental supports.

## Conflicts of Interest

The authors declare no conflicts of interest.

## Supporting information




**Supporting File 1**: advs77077‐sup‐0001‐SuppMat.docx.

**Supporting File 2**: advs77077‐sup‐0002‐FigureS1‐S9.zip.

**Supporting File 3**: advs77077‐sup‐0003‐TableS1‐S3.zip.

## Data Availability

No written informed consent was obtained from the patients as no identifiable patient data was included, and therefore no clinical study registration number applies. The supporting data are available within the article and its supplementary materials.
