# Supplementary material for: OTUD6A‐Mediated Deubiquitination of PRDX1 Protects Against Oral Ulcer by Restoring Mitochondrial Function
Source: Adv Sci (Weinh). 2026 Aug 12;13(61):e77077. doi: 10.1002/advs.77077 (PMC13464554; doi:10.1002/advs.77077)
Supplement: Supplementary file 3 [file ADVS-13-e77077-s003.docx]

**OTUD6A-Mediated Deubiquitination of PRDX1 Protects Against Oral Ulcer by Restoring Mitochondrial Function**

***Supplementary Information***

Supplementary Information Contents:

1. Figure S1-S9
2. Table S1-S3

***Supplementary Figures:***

**Figure S1. The general phenotypes of *Otud6a^-/^*^-^ and *Otud6a*^+/+^ mice.** (A) Genotyping of *Otud6a^-/-^* mice. Genomic DNA was extracted from mouse tails and analyzed by PCR followed by agarose gel electrophoresis. The mutant (*Otud6a^-/-^*) allele and wild-type (*Otud6a^+/+^*) allele yielded products of ~335 bp and ~516 bp, respectively. (B, C) Representative western blot and quantitative analysis of OTUD6A protein expression in mouse tongue tissues, n = 6 per group. (D) Tongue tissue morphology from *Otud6a^+/+^* and Otu*d6a^-/-^* mice, n = 3 per group. Scale bar = 400 μm. ***p < 0.001.

**Figure S2. OTUD6A deubiquitinated PRDX1.** (A) PRDX1 protein levels in ulcer and non-ulcer tissues from mice with OU, n = 3 per group. Scale bar = 100 μm. (B) Schematic diagram of OTUD6A and its truncated mutants. (C) Schematic illustration of the PRDX1 domain deletion constructs. (D) Results of ubiquitination assays confirming the ubiquitination of PRDX1 after overexpression of *OTUD6A* and *PRDX1* for 24 h and treated with 10 μM MG132 for 6 h in HEK-293T cells, n = 3 per group. (E) Immunoprecipitation of PRDX1 in HEK-293T cells co-transfected with GFP-PRDX1, FLAG-OTUD6A, and HA-UB, HA-UB-K48 (K48 only), HA-UB-K63 (K63 only) and then challenged with 10 μM MG132 for 6 h. Ubiquitinated PRDX1 detected by immunoblotting staining using an anti-HA antibody, n = 3 per group. (F) Ubiquitination of lysine residues in the potential substrate PRDX1 was revealed through GPS-Uber analysis. (G-I) Immunoprecipitation of PRDX1 in HEK-293T cells co-transfected with FLAG-OTUD6A, HA-UB, GFP-PRDX1, GFP-PRDX1^K7R^, GFP-PRDX1^K16R^, and GFP-PRDX1^K120R^ plasmids. Exogenous ubiquitinated PRDX1 was detected by immunoblotting using a GFP-specific antibody to identify the specific lysine residues on PRDX1 responsible for its ubiquitination, n = 3 per group.

**Figure S3. PRDX1 attenuated mitochondrial oxidative stress and dysfunction of HOKs.** (A) MTT assay to quantify cell viability. Data are presented as mean ± SEM, n =4 per group. (B, C) Apoptosis rate of HOKs determined by transferase-mediated dUTP nick end labeling (TUNEL) assay and expressed as the percentage of apoptotic HOKs. Data are presented as mean ± SEM, n = 3 per group. Scale bar = 200 μm. (D, E) HOKs migration as analyzed by scratch wound assay. Data are presented as mean ± SEM, n = 3 per group. Scale bar = 200 μm. (F, G) Representative 2',7'-Dichlorodihydrofluorescein diacetate (DCFH-DA) staining images (F) and quantitative analysis (G) of HOKs upon different treatments. Data are presented as mean ± SEM, n = 3 per group. Scale bar = 100 μm. (H) The ATP content of HOKs upon different treatments. Data are presented as mean ± SEM, n = 3 per group. (I, J) Representative Mitosox staining images (I) and quantitative analysis (J) of HOKs upon different treatments. Data are presented as mean ± SEM, n = 3 per group. Scale bar = 100 μm. (K, L) Representative TMRM staining images (K) and quantitative analysis (L) of HOKs upon different treatments. Data are presented as mean ± SEM, n = 3 per group. Scale bar = 100 μm. *p < 0.05, **p < 0.01, ***p < 0.001.

**Figure S4. OTUD6A attenuated mitochondrial oxidative stress and dysfunction of HOKs.** (A) Representative DCFH-DA staining images of HOKs transfected with or without *Si-OTUD6A* (or *Si-NC*) after H_2_O_2_ exposure. n*=* 3 per group. Scale bar = 100 μm. (B) Representative Mitosox staining images analysis of HOKs transfected with or without *Si-OTUD6A* (or *Si-NC*) after H_2_O_2_ exposure, n = 3 per group. Scale bar = 100 μm. (C) Representative TMRM staining images of HOKs transfected with or without *Si-OTUD6A* (or *Si-NC*) after H_2_O_2_ exposure, n = 3 per group. Scale bar = 100 μm. (D-F) Quantitative analysis of DCFH-DA, Mitosox, and TMRM staining of HOKs transfected with or without *Si-OTUD6A* (or *Si-NC*) after H_2_O_2_ exposure. Data are presented as mean ± SEM, n = 3 per group. (G) ATP content of HOKs transfected with or without *Si-OTUD6A* (or *Si-NC*) after H_2_O_2_ exposure. Data are presented as mean ± SEM, n = 6 per group. (H) Representative DCFH-DA staining images of HOKs transfected with or without *OTUD6A^oe^* (or EV) after H_2_O_2_ exposure, n = 3 per group. Scale bar = 100 μm. (I) Representative Mitosox staining images of HOKs transfected with or without *OTUD6A^oe^* (or EV) after H_2_O_2_ exposure, n = 3 per group. Scale bar = 100 μm. (J) Representative TMRM staining images of HOKs transfected with or without *OTUD6A^oe^* (or EV) after H_2_O_2_ exposure. n = 3 per group. Scale bar = 100 μm. (K-M) Quantitative analysis of DCFH-DA, Mitosox, and TMRM staining of HOKs transfected with or without *OTUD6A^oe^* (or EV) after H_2_O_2_ exposure. Data are presented as mean ± SEM, n = 3 per group. (N) ATP content of HOKs transfected with or without *OTUD6A^oe^* (or EV) after H_2_O_2_ exposure. Data are presented as mean ± SEM, n = 4 per group. *p < 0.05, **p < 0.01, ***p < 0.001.

**Figure S5. MitoQ treatment alleviated apoptosis and migration impairment induced by OTUD6A knockdown in HOKs under oxidative stress.** (A) Cell viability was quantified using the MTT assay. Data are presented as mean ± SEM, n = 4 per group. (B, C) Apoptosis of HOKs was determined by TUNEL assay (B) and expressed quantitatively as the TUNEL positive rate relative to the control (C). Data are presented as mean ± SEM, n = 3 per group. Scale bar = 200 μm. (D, E) The migration capacity of HOKs was analyzed by scratch wound assay at 0 h and 24 h. (D) and quantitatively expressed as the migration rate relative to the control (E). Data are presented as mean ± SEM, n = 3 per group. Scale bar = 200 μm. *p < 0.05, **p < 0.01, ***p< 0.001.

**Figure S6. OTUD6A deficiency increased 8-OHDG expression in mouse tongue tissue.** (A) Representative immunohistochemistry images of 8-OHDG in tongue tissues from *Otud6a^+/+^* mice and *Otud6a^-/-^* mice with or without OU, n = 6 per group. Scale bar = 500 μm (Enlarged image scale bar = 100 μm). (B) ATP levels in ulcerated tongue tissues from *Otud6a^+/+^* and *Otud6a^-/-^* mice. Data are presented as mean ± SEM, n = 6 per group. ***p < 0.001.

**Figure S7. OTUD6A modulated the function and stability of PRDX1.** (A) Quantitative analysis of blots in Figure. 4A. Data are presented as mean ± SEM, n = 3 per group. (B, C) Representative immunoblot (B) and densitometric quantification (C) of PRDX1 in control or *OTUD6A*-overexpressing HEK-293T cells subjected to a CHX pulse-chase assay. Data are presented as mean ± SEM, n = 3 per group. (D) Representative immunohistochemistry images of PRDX1 in tongue tissues of *Otud6a^+/+^* mice and *Otud6a^-/-^* mice. Scale bar = 100 μm. (E, F) Densitometric quantification of blots in Figure. 4C. Data are presented as mean ± SEM, n = 3 per group. (G, H) Densitometric quantification of blots in Figure. 4D. n = 3 per group. (I) Quantitative analysis of DCFH-DA staining in Figure. 4J. Data are presented as mean ± SEM, n = 3 per group. (J) Quantitative analysis of Mitosox staining in Figure. 4L. Data are presented as mean ± SEM, n = 3 per group. (K) Quantitative analysis of TMRM staining in Figure. 4M. Data are presented as mean ± SEM, n = 3 per group. *p < 0.05, **p < 0.01, ***p < 0.001.

**Figure S8. PRDX1 knockdown abolished the protective effects of OTUD6A overexpression against H_2_O_2_-induced injury in HOKs.** (A, B) Representative immunoblots and quantitative analysis of PRDX1 knockdown efficiency in HOKs. Three independent siRNAs (*Si-PRDX1-1*, *Si-PRDX1-2*, *Si-PRDX1-3*) were used, with *Si-NC* as a negative control. GAPDH was used as the loading control, n = 3 per group. (C) Cell viability was quantified using the MTT assay. Data are presented as mean ± SEM, n = 4 per group. (D, E) Apoptosis of HOKs was determined by TUNEL assay (D) and expressed quantitatively as the TUNEL positive rate relative to the control (E). Data are presented as mean ± SEM, n = 3 per group. Scale bar = 200 μm. (F, G) Migration of HOKs was analyzed by scratch wound assay at 0 h and 24 h (F) and expressed quantitatively as the migration rate relative to the control (G). Data are presented as mean ± SEM, n = 3 per group. Scale bar = 200 μm. *p < 0.05, **p < 0.01, ***p < 0.001.

**Figure S9. Characterization of OTUD6A@HmA and its therapeutic effect on oral ulcer (OU) healing**. (A) Preparation of HmA and OTUD6A@HmA solutions: (1) His6 solution without Zn^2+^; (2) HmA solution with Zn^2+^; (3) OTUD6A-His6 solution without Zn^2+^; (4) OTUD6A@HmA solution with Zn^2+^. The Tyndall effect observed in (2) and (4) indicates the successful formation of nanoparticles. (B, C) Morphological characterization of nanoparticles by SEM (B) and TEM (C). Scale bar = 100 nm. (D, E) Particle size distribution (D) and Zeta potential (E) of the nanoparticles. (F–I) Stability assessments showing size (F) and PDI across different batches (G), and size (H) and PDI (I) over 7 days. (J) Standard curve for OTUD6A quantification. Absorbance at 280 nm (a.u.) versus OTUD6A concentration (mg/mL). Linear regression: Y = 0.992*x + 0.2008, R² = 0.9579. Data are presented as mean ± SEM, n = 3 per group. (K) Cumulative release rate of OTUD6A from the nanoparticles at different pH levels. (L–N) Quantitative analysis corresponding to the treatments, including macroscopic scores (L), OU depth (M), and body weight changes (N), n = 8 per group. (O, P) Representative immunohistochemical images illustrating the expression of OTUD6A (O) and PRDX1 (P) in paraffin-embedded oral ulcer tissue sections from the sham and treated mice, n = 8 per group. Scale bar = 500 μm (Enlarged image scale bar = 50 μm). **p < 0.01, ***p < 0.001; ns, no significance.

***Supplementary Tables:***

**Table S1.** 4 lysine residues could be potentially ubiquitinated, according to the prediction by the GPS-Uber (http://gpsuber.biocuckoo.cn).

| Position | Peptide | Score |
| --- | --- | --- |
| 7 | ****MSSGNAKIGYPAPNEKA | 0.6117 |
| 16 | AKIGYPAPNEKATAVMPDGQE | 0.4109 |
| 120 | RTIAODYGVLKADEGISFRGL | 0.7077 |
| 136 | SPRGLPIIDDKGILRQITIND | 0.4502 |

**Table S2.** Sequences of primers for RT-qPCR assay used in the study.

| **Gene** | **Species** | **FW** | **RW** |
| --- | --- | --- | --- |
| *Actin* | Mouse | CCGTGAAAAGATGACCCAGA | TACGACCAGAGGCATACAG |
| *Yod1* | Mouse | AAGTCTCCCTGTGTGTTTCTTGTCG | ACTCAGAAGGCAGAGGCAGAAGG |
| *Alg13* | Mouse | TGCCCAAACCATTCCGTACTGAG | TGCCTTTCTCCAGACTCTCCAAAC |
| *Otud1* | Mouse | CTCTGCCTGGCTGCTGGAAG | GGTGCTCGCTCAGTCGGAAG |
| *Otud3* | Mouse | CGGCTGCGAAGAAGAGTTTGTG | TGGCGATGCTTGAGATGGTTCC |
| *Otud4* | Mouse | ACTCCTGCGGTGCCTTCTTTAC | CGGCAGCATCAGGTCCAGTG |
| *Otud5* | Mouse | TGCCCAAACCATTCCGTACTGAG | TGCCTTTCTCCAGACTCTCCAAAC |
| *Otud6a* | Mouse | TGTCACCAACGCTCCAAGTCTG | TTGCTTGTCCCTGCTCTGTCTC |
| *Otud6b* | Mouse | AGGTGACTTAGCAGCCATAGAAGC | AGCAGAAGGACGGTTCAGCAAG |
| *Otud7a* | Mouse | ACAGCAGAACAAGGAGGAGGAATG | GAGTTGTCCACACCACCACCTG |
| *Otud7b* | Mouse | CACCAACGAAGAGGAGGAGTACAG | GGGCAAACAAGAGCACAGAGAAG |
| *Otub1* | Mouse | GCCCTCAGTGTGTCCATCCAAG | TCGTAGTGTCCAGGTCGGTAGAG |
| *Otub2* | Mouse | ACCTCATTCCTCGCTTCCATCTG | AGTGGGTAAGACAAGACGGAGAAC |
| *Otulin* | Mouse | CACCAACGAAGAGGAGGAGTACAG | GGGCAAACAAGAGCACAGAGAAG |
| *Prdx1* | Mouse | TGCCAAGTGATTGGCGCTTCTG | AGCAATGGTGCGCTTGGGATCT |
| *Tnfaip3* | Mouse | TGTCACCAACGCTCCAAGTCTG | TTGCTTGTCCCTGCTCTGTCTC |
| *Zranb1* | Mouse | AGGTGACTTAGCAGCCATAGAAGC | AGCAGAAGGACGGTTCAGCAAG |

**Table S3.** List of TOP 20 OTUD6A-interacting proteins identified by LC-MS/MS.

| **NO.** | **Protein accession** | **Protein description** | **Gene name** | **Intensity**  **OTUD6A/Control Ratio** | **Coverage rate [%]** | **Unique peptides** | **# PSMs** | **Razor + unique peptides** | **Razor + unique peptides Control** | **Razor + unique peptides OTUD6A** | **iBAQ Control** | **iBAQ OTUD6A** |
| --- | --- | --- | --- | --- | --- | --- | --- | --- | --- | --- | --- | --- |
| 1 | Q7L8S5 | OTU domain-containing protein 6A OS=Homo sapiens OX=9606 GN=OTUD6A PE=1 SV=1 | OTUD6A | 1000 | 75.2 | 17 | 1 | 17 | 1 | 17 | — | 2004300000 |
| 2 | P61366 | Osteocrin OS=Homo sapiens OX=9606 GN=OSTN PE=1 SV=1 | OSTN | 15.1 | 79.6 | 12 | 1 | 11 | 11 | 11 | 22071000 | 283240000 |
| 3 | Q06830 | Peroxiredoxin-1 OS=Homo sapiens OX=9606 GN=PRDX1 PE=1 SV=1 | PRDX1 | 14.6 | 76.5 | 16 | 2 | 7 | 5 | 7 | 20058000 | 304230000 |
| 4 | Q8NBI6 | Xyloside xylosyltransferase 1 OS=Homo sapiens OX=9606 GN=XXYLT1 PE=1 SV=1 | XXYLT1 | 12.3 | 70.6 | 9 | 1 | 4 | 3 | 4 | 20032400 | 298790000 |
| 5 | Q9BXM7 | Serine/threonine-protein kinase PINK1, mitochondrial OS=Homo sapiens OX=9606 GN=PINK1 PE=1 SV=2 | PINK1 | 10.9 | 69.5 | 10 | 1 | 3 | 3 | 3 | 21062000 | 294550000 |
| 6 | P08670 | Vimentin OS=Homo sapiens OX=9606 GN=VIM PE=1 SV=3 | VIM | 10.811 | 59 | 28 | 1 | 28 | 16 | 28 | 20058000 | 314456800 |
| 7 | P05386 | 60S acidic ribosomal protein P1 OS=Homo sapiens OX=9606 GN=RPLP1 PE=1 SV=1 | RPLP1 | 3.886 | 59.8 | 4 | 1 | 4 | 4 | 4 | 168110000 | 475450000 |
| 8 | P60002 | Transcription elongation factor 1 homolog OS=Homo sapiens OX=9606 GN=ELOF1 PE=1 SV=1 | ELOF1 | 3.267 | 69.9 | 2 | 1 | 2 | 2 | 1 | 88660000 | 120440000 |
| 9 | P62304 | Small nuclear ribonucleoprotein E OS=Homo sapiens OX=9606 GN=SNRPE PE=1 SV=1 | SNRPE | 2.073 | 65.2 | 4 | 1 | 4 | 4 | 4 | 61245000 | 78378000 |
| 10 | P68371 | Tubulin beta-4B chain OS=Homo sapiens OX=9606 GN=TUBB4B PE=1 SV=1 | TUBB4B | 1.982 | 61.8 | 2 | 2 | 3 | 3 | 3 | 32570000 | 23320000 |
| 11 | P05387 | 60S acidic ribosomal protein P2 OS=Homo sapiens OX=9606 GN=RPLP2 PE=1 SV=3 | RPLP2 | 1.931 | 69.6 | 5 | 1 | 5 | 5 | 5 | 273490000 | 493390000 |
| 12 | P60660 | Myosin light polypeptide 6 OS=Homo sapiens OX=9606 GN=MYL6 PE=1 SV=3 | MYL6 | 1.872 | 55.6 | 8 | 1 | 8 | 8 | 8 | 313790000 | 497430000 |
| 13 | P30050 | 60S ribosomal protein L12 OS=Homo sapiens OX=9606 GN=RPL12 PE=1 SV=2 | RPL12 | 1.864 | 64.8 | 8 | 1 | 8 | 6 | 7 | 272880000 | 357070000 |
| 14 | P55769 | NHP2-like protein 1 OS=Homo sapiens OX=9606 GN=SNU13 PE=1 SV=4 | SNU13 | 1.796 | 68 | 7 | 1 | 7 | 5 | 7 | 60765000 | 184360000 |
| 15 | P84090 | Enhancer of rudimentary homolog OS=Homo sapiens OX=9606 GN=ERH PE=1 SV=1 | ERH | 1.786 | 75 | 5 | 1 | 5 | 4 | 5 | 612840000 | 776460000 |
| 16 | P05388 | 60S acidic ribosomal protein P0 OS=Homo sapiens OX=9606 GN=RPLP0 PE=1 SV=3 | RPLP0 | 1.728 | 55.8 | 12 | 1 | 12 | 12 | 11 | 221860000 | 214520000 |
| 17 | P63261 | Actin, cytoplasmic 2 OS=Homo sapiens OX=9606 GN=ACTG1 PE=1 SV=1 | ACTG1 | 1.536 | 61.1 | 1 | 1 | 18 | 15 | 18 | 861560000 | 1468700000 |
| 18 | P68363 | Tubulin alpha-1B chain OS=Homo sapiens OX=9606 GN=TUBA1B PE=1 SV=2 | TUBA1B | 0.631 | 70.3 | 0 | 1 | 20 | 19 | 16 | 408900000 | 251190000 |
| 19 | P62081 | 40S ribosomal protein S7 OS=Homo sapiens OX=9606 GN=RPS7 PE=2 SV=1 | RPS7 | 0.432 | 55.7 | 10 | 1 | 10 | 10 | 9 | 1365300000 | 664490000 |
| 20 | Q71U36 | Tubulin alpha-1A chain OS=Homo sapiens OX=9606 GN=TUBA1A PE=1 SV=1 | TUBA1A | 0.206 | 70.3 | 1 | 2 | 2 | 2 | 2 | 40267000 | 5602200 |
