# Supplementary material for: OTUD6A‐Mediated Deubiquitination of PRDX1 Protects Against Oral Ulcer by Restoring Mitochondrial Function
Source: Adv Sci (Weinh). 2026 Aug 12:e77077. Online ahead of print. doi: 10.1002/advs.77077 (PMC13464554; doi:10.1002/advs.77077)
Supplement: Supplementary file 1 [file ADVS-9999-e77077-s001.zip › Table S1.docx]

**Table S1:** 4 lysine residues could be potentially ubiquitinated, according to the prediction by the GPS-Uber (http://gpsuber.biocuckoo.cn).

| Position | Peptide | Score |
| --- | --- | --- |
| 7 | ****MSSGNAKIGYPAPNEKA | 0.6117 |
| 16 | AKIGYPAPNEKATAVMPDGQE | 0.4109 |
| 120 | RTIAODYGVLKADEGISFRGL | 0.7077 |
| 136 | SPRGLPIIDDKGILRQITIND | 0.4502 |
