# Supplementary material for: OTUD6A‐Mediated Deubiquitination of PRDX1 Protects Against Oral Ulcer by Restoring Mitochondrial Function
Source: Adv Sci (Weinh). 2026 Aug 12:e77077. Online ahead of print. doi: 10.1002/advs.77077 (PMC13464554; doi:10.1002/advs.77077)
Supplement: Supplementary file 1 [file ADVS-9999-e77077-s001.zip › Table S2.docx]

**Table S2:** Sequences of primers for RT-qPCR assay used in the study.

| **Gene** | **Species** | **FW** | **RW** |
| --- | --- | --- | --- |
| *Actin* | Mouse | CCGTGAAAAGATGACCCAGA | TACGACCAGAGGCATACAG |
| *Yod1* | Mouse | AAGTCTCCCTGTGTGTTTCTTGTCG | ACTCAGAAGGCAGAGGCAGAAGG |
| *Alg13* | Mouse | TGCCCAAACCATTCCGTACTGAG | TGCCTTTCTCCAGACTCTCCAAAC |
| *Otud1* | Mouse | CTCTGCCTGGCTGCTGGAAG | GGTGCTCGCTCAGTCGGAAG |
| *Otud3* | Mouse | CGGCTGCGAAGAAGAGTTTGTG | TGGCGATGCTTGAGATGGTTCC |
| *Otud4* | Mouse | ACTCCTGCGGTGCCTTCTTTAC | CGGCAGCATCAGGTCCAGTG |
| *Otud5* | Mouse | TGCCCAAACCATTCCGTACTGAG | TGCCTTTCTCCAGACTCTCCAAAC |
| *Otud6a* | Mouse | TGTCACCAACGCTCCAAGTCTG | TTGCTTGTCCCTGCTCTGTCTC |
| *Otud6b* | Mouse | AGGTGACTTAGCAGCCATAGAAGC | AGCAGAAGGACGGTTCAGCAAG |
| *Otud7a* | Mouse | ACAGCAGAACAAGGAGGAGGAATG | GAGTTGTCCACACCACCACCTG |
| *Otud7b* | Mouse | CACCAACGAAGAGGAGGAGTACAG | GGGCAAACAAGAGCACAGAGAAG |
| *Otub1* | Mouse | GCCCTCAGTGTGTCCATCCAAG | TCGTAGTGTCCAGGTCGGTAGAG |
| *Otub2* | Mouse | ACCTCATTCCTCGCTTCCATCTG | AGTGGGTAAGACAAGACGGAGAAC |
| *Otulin* | Mouse | CACCAACGAAGAGGAGGAGTACAG | GGGCAAACAAGAGCACAGAGAAG |
| *Prdx1* | Mouse | TGCCAAGTGATTGGCGCTTCTG | AGCAATGGTGCGCTTGGGATCT |
| *Tnfaip3* | Mouse | TGTCACCAACGCTCCAAGTCTG | TTGCTTGTCCCTGCTCTGTCTC |
| *Zranb1* | Mouse | AGGTGACTTAGCAGCCATAGAAGC | AGCAGAAGGACGGTTCAGCAAG |
