# Supplementary material for: OTUD6A‐Mediated Deubiquitination of PRDX1 Protects Against Oral Ulcer by Restoring Mitochondrial Function
Source: Adv Sci (Weinh). 2026 Aug 12:e77077. Online ahead of print. doi: 10.1002/advs.77077 (PMC13464554; doi:10.1002/advs.77077)
Supplement: Supplementary file 1 [file ADVS-9999-e77077-s001.zip › Table S3.docx]

**Table S3.** List of TOP 20 OTUD6A-interacting proteins identified by LC-MS/MS.

| **NO.** | **Protein accession** | **Protein description** | **Gene name** | **Intensity** **OTUD6A/Control Ratio** | **Coverage rate [%]** | **Unique peptides** | **# PSMs** | **Razor + unique peptides** | **Razor + unique peptides Control** | **Razor + unique peptides OTUD6A** | **iBAQ Control** | **iBAQ OTUD6A** |
| --- | --- | --- | --- | --- | --- | --- | --- | --- | --- | --- | --- | --- |
| 1 | Q7L8S5 | OTU domain-containing protein 6A OS=Homo sapiens OX=9606 GN=OTUD6A PE=1 SV=1 | OTUD6A | 1000 | 75.2 | 17 | 1 | 17 | 1 | 17 | — | 2004300000 |
| 2 | P61366 | Osteocrin OS=Homo sapiens OX=9606 GN=OSTN PE=1 SV=1 | OSTN | 15.1 | 79.6 | 12 | 1 | 11 | 11 | 11 | 22071000 | 283240000 |
| 3 | Q06830 | Peroxiredoxin-1 OS=Homo sapiens OX=9606 GN=PRDX1 PE=1 SV=1 | PRDX1 | 14.6 | 76.5 | 16 | 2 | 7 | 5 | 7 | 20058000 | 304230000 |
| 4 | Q8NBI6 | Xyloside xylosyltransferase 1 OS=Homo sapiens OX=9606 GN=XXYLT1 PE=1 SV=1 | XXYLT1 | 12.3 | 70.6 | 9 | 1 | 4 | 3 | 4 | 20032400 | 298790000 |
| 5 | Q9BXM7 | Serine/threonine-protein kinase PINK1, mitochondrial OS=Homo sapiens OX=9606 GN=PINK1 PE=1 SV=2 | PINK1 | 10.9 | 69.5 | 10 | 1 | 3 | 3 | 3 | 21062000 | 294550000 |
| 6 | P08670 | Vimentin OS=Homo sapiens OX=9606 GN=VIM PE=1 SV=3 | VIM | 10.811 | 59 | 28 | 1 | 28 | 16 | 28 | 20058000 | 314456800 |
| 7 | P05386 | 60S acidic ribosomal protein P1 OS=Homo sapiens OX=9606 GN=RPLP1 PE=1 SV=1 | RPLP1 | 3.886 | 59.8 | 4 | 1 | 4 | 4 | 4 | 168110000 | 475450000 |
| 8 | P60002 | Transcription elongation factor 1 homolog OS=Homo sapiens OX=9606 GN=ELOF1 PE=1 SV=1 | ELOF1 | 3.267 | 69.9 | 2 | 1 | 2 | 2 | 1 | 88660000 | 120440000 |
| 9 | P62304 | Small nuclear ribonucleoprotein E OS=Homo sapiens OX=9606 GN=SNRPE PE=1 SV=1 | SNRPE | 2.073 | 65.2 | 4 | 1 | 4 | 4 | 4 | 61245000 | 78378000 |
| 10 | P68371 | Tubulin beta-4B chain OS=Homo sapiens OX=9606 GN=TUBB4B PE=1 SV=1 | TUBB4B | 1.982 | 61.8 | 2 | 2 | 3 | 3 | 3 | 32570000 | 23320000 |
| 11 | P05387 | 60S acidic ribosomal protein P2 OS=Homo sapiens OX=9606 GN=RPLP2 PE=1 SV=3 | RPLP2 | 1.931 | 69.6 | 5 | 1 | 5 | 5 | 5 | 273490000 | 493390000 |
| 12 | P60660 | Myosin light polypeptide 6 OS=Homo sapiens OX=9606 GN=MYL6 PE=1 SV=3 | MYL6 | 1.872 | 55.6 | 8 | 1 | 8 | 8 | 8 | 313790000 | 497430000 |
| 13 | P30050 | 60S ribosomal protein L12 OS=Homo sapiens OX=9606 GN=RPL12 PE=1 SV=2 | RPL12 | 1.864 | 64.8 | 8 | 1 | 8 | 6 | 7 | 272880000 | 357070000 |
| 14 | P55769 | NHP2-like protein 1 OS=Homo sapiens OX=9606 GN=SNU13 PE=1 SV=4 | SNU13 | 1.796 | 68 | 7 | 1 | 7 | 5 | 7 | 60765000 | 184360000 |
| 15 | P84090 | Enhancer of rudimentary homolog OS=Homo sapiens OX=9606 GN=ERH PE=1 SV=1 | ERH | 1.786 | 75 | 5 | 1 | 5 | 4 | 5 | 612840000 | 776460000 |
| 16 | P05388 | 60S acidic ribosomal protein P0 OS=Homo sapiens OX=9606 GN=RPLP0 PE=1 SV=3 | RPLP0 | 1.728 | 55.8 | 12 | 1 | 12 | 12 | 11 | 221860000 | 214520000 |
| 17 | P63261 | Actin, cytoplasmic 2 OS=Homo sapiens OX=9606 GN=ACTG1 PE=1 SV=1 | ACTG1 | 1.536 | 61.1 | 1 | 1 | 18 | 15 | 18 | 861560000 | 1468700000 |
| 18 | P68363 | Tubulin alpha-1B chain OS=Homo sapiens OX=9606 GN=TUBA1B PE=1 SV=2 | TUBA1B | 0.631 | 70.3 | 0 | 1 | 20 | 19 | 16 | 408900000 | 251190000 |
| 19 | P62081 | 40S ribosomal protein S7 OS=Homo sapiens OX=9606 GN=RPS7 PE=2 SV=1 | RPS7 | 0.432 | 55.7 | 10 | 1 | 10 | 10 | 9 | 1365300000 | 664490000 |
| 20 | Q71U36 | Tubulin alpha-1A chain OS=Homo sapiens OX=9606 GN=TUBA1A PE=1 SV=1 | TUBA1A | 0.206 | 70.3 | 1 | 2 | 2 | 2 | 2 | 40267000 | 5602200 |
